# Thymic stromal lymphopoietin expression in different biological specimens in asthma: a systematic review and meta-analysis

**DOI:** 10.3389/falgy.2025.1703989

**Published:** 2025-11-20

**Authors:** Izabela Orzołek, Patrycja Kowalczyk, Aleksandra Rybka-Frączek, Monika Storman, Marta Dąbrowska

**Affiliations:** 1 Doctoral School of Medical University of Warsaw, Warsaw, Poland; 2 Department of Internal Medicine, Pulmonary Diseases and Allergy, Medical University of Warsaw, Warsaw, Poland; 3 Students’ Research Group “Alveolus”, Warsaw, Poland

**Keywords:** TSLP, thymic stromal lymphopoietin, asthma, systematic review, meta-analysis

## Abstract

**Background:**

Thymic stromal lymphopoietin (TSLP) is an epithelial-derived cytokine implicated in the pathogenesis of asthma. However, its expression across different biological specimens and its clinical correlates remain unclear. The objective of this study was to summarize available data on TSLP concentrations in blood and airway specimens in asthmatic patients.

**Methods:**

Studies reporting TSLP concentrations in blood and/or airway specimens [e.g., bronchial biopsy, bronchoalveolar lavage fluid (BALf), induced sputum, exhaled breath condensate (EBC), and nasal specimens] of asthmatic patients compared with healthy controls were eligible. PubMed, Web of Science, Embase, and Cochrane Library were searched from inception to October 2024. A total of 40 studies were included for qualitative synthesis, and 5 were eligible for meta-analysis. Differences in TSLP levels of asthmatic patients and controls were summarized by standardized mean differences (SMD) using a random effects model.

**Results:**

Based on meta-analysis, blood TSLP concentration was significantly higher in patients with asthma than in controls (SMD = 3.66, 95% CI 1.63–5.69, *I*^2^ = 98.26%). The sensitivity analysis showed that no individual study influenced the pooled effect estimate. Based on a systematic review, all studies analyzing bronchial biopsies and BALf reported significantly higher TSLP concentration in asthmatics compared with controls, whereas results in induced sputum, EBC, and nasal specimens were variable.

**Conclusions:**

Most studies reported higher blood TSLP concentration in asthma patients compared with healthy controls, while results in airway specimens were diverse. Higher concentration of TSLP in asthmatic patients might be a useful disease-related marker.

**Systematic Review Registration:**

https://www.crd.york.ac.uk/PROSPERO/view/CRD42024537964, PROSPERO CRD42024537964.

## 1 Introduction

Asthma affects around 300 million people worldwide, being a major non-communicable disease and a global healthcare burden (1). Asthma is a heterogenous disease characterized by airway hyperresponsiveness and airway inflammation, contributing to variable airflow obstruction (1).

The airway epithelium is increasingly recognized as playing a key role in dysregulated immune responses in asthma (2). Disrupted airway epithelium is ineffective in protecting against inhaled particles, e.g., allergens, smoke, and viruses, and increases their penetration to the airways (3). Stimulated airway epithelial cells release epithelium-derived cytokines—thymic stromal lymphopoietin (TSLP), IL-33, and IL-25—representing the top of the immunologic cascade, further commencing both T2-high and T2-low responses (4, 5).

In the lungs, TSLP is predominantly produced by activated bronchial epithelial cells, airway smooth muscle cells, lung macrophages, dendritic cells (DCs), and mast cells (6). TSLP activates intracellular signaling by binding to a complex receptor composed of the TSLP receptor (TSLPR) and interleukin-7 receptor alpha chain, activating downstream signaling pathways (5). TSLP-activated dendritic cells stimulate naive CD4+ T cells differentiation into T2 inflammatory cells (7).

A pair of TSLP isoforms has been identified—long form TSLP (lfTSLP) and short form TSLP (sfTSLP), each playing distinct immunological roles (5). The long isoform of TSLP is upregulated in inflammatory conditions and promotes T2 immune responses (4, 8). Conversely, the short isoform of TSLP is expressed constitutively and is considered to play a homeostatic, anti-inflammatory role (5). Previous studies showed that high concentrations of sfTSLP can inhibit the disruption of the epithelium induced by lfTSLP (9). In mice, sfTSLP was observed to reduce airway inflammation induced by house dust mite and lfTSLP (8). However, the biological implications of both isoforms in the biology of asthma remain unclear.

Tezepelumab is the first monoclonal antibody blocking the biological activity of TSLP approved for patients with T2-high or T2-low phenotype of severe asthma (10), whereas all previously approved biological agents are indicated only for T2-high asthma. In clinical trials, tezepelumab showed effectiveness in reducing asthma exacerbation rates, improving lung function and quality of life, irrespective of baseline eosinophil counts (11). Currently, several clinical trials examine the efficacy and safety of tezepelumab in other disorders, including severe chronic rhinosinusitis with nasal polyps, chronic spontaneous urticaria, and chronic obstructive pulmonary disease.

The association between plasma TSLP concentrations with asthma status and asthma severity is inconsistent, with some studies reporting elevated concentrations of serum TSLP in asthmatic subjects compared with healthy individuals. To date, most experimental studies evaluating TSLP in asthma do not use analytic tools to measure expression of short and long TSLP isoforms separately.

The validity of measuring systemic or local expression of TSLP in the diagnosis or in the management of asthma remains unclear. The objective of this systematic review and meta-analysis is to summarize existing data on the difference in TSLP concentration or expression in biological specimens of asthmatic patients compared with healthy controls.

## 2 Methods

### 2.1 Study protocol

This systematic review and meta-analysis were conducted according to the Preferred Reporting Items for Systematic Reviews and Meta-Analyses (PRISMA) 2020 checklist to ensure quality and transparency (12). The study protocol was registered on the International Prospective Registry of Systematic Reviews (PROSPERO CRD42024537964). No ethics committee approval was required for this study.

### 2.2 Search strategy

We searched electronic databases (PubMed, Web of Science, Embase, and Cochrane Library) for articles published from their inception until 22 April 2024. The update search was performed on 22 October 2024. The search strategy for each database is detailed in the Supplementary Material. Reference lists were checked for any additional relevant studies.

### 2.3 Eligibility criteria (inclusion and exclusion criteria)

Studies were included based on the predefined criteria:

1. Study design: original studies including cohort, case–control, and cross-sectional studies
2. Participants: studies enrolling individuals diagnosed with asthma aged over 1 year old, without an upper age limit
3. Control: studies involving a group of healthy controls without any obstructive diseases or other chronic pathologies
4. TSLP expression: studies reporting data on TSLP mRNA or protein expression levels in serum or airway specimens, e.g., bronchial biopsy, induced sputum, bronchial washing, bronchoalveolar lavage fluid (BALf), and nasal specimens, measured in both asthmatics and healthy controls, with no restriction to the laboratory technique
5. Language: studies published in English

We excluded articles lacking numerical or graphical data on TSLP concentrations in asthmatic patients and healthy controls, not available in the English language, enrolled populations of patients below 1 year of age, or without a group of healthy controls. Additionally, studies assessing TSLP levels only in cell cultures or non-human subjects were excluded. In cases where multiple studies reported the same dataset, the most recent and comprehensive version was included in the meta-analysis. We excluded non-English articles to ensure consistency in methodology and avoid language bias.

### 2.4 Study selection

All extracted citations were imported into the EndNote® reference manager (Version X9, Clarivate Analytics, 2018), where duplicates were excluded. Two reviewers (IO and PK) independently screened the retrieved titles and abstracts in accordance with pre-established eligibility criteria. Subsequently, the same reviewers assessed all potentially relevant publications in full text. Any discrepancies at any stage of the screening were resolved through discussion with the third author uninvolved in the initial screening (MD).

### 2.5 Data extraction

We extracted details of study origin, study design, sample size, population (sex, age, asthma severity, BMI, presence of atopy, respiratory parameters), type of biological material for analysis [e.g., blood, bronchial biopsy, induced sputum, BALf, exhaled breath condensate (EBC), and nasal specimens], mean and standard deviation (SD), or median and interquartile range (IQR) of TSLP expression level (pg/mL) in asthma groups and in healthy controls. We also sought any additional outcomes, including the relationship between TSLP concentrations and various factors, including age, sex, atopic characteristics (allergies, atopic dermatitis), family history of allergy/asthma, lung function parameters (FEV1, FVC), asthma control, and total serum IgE concentration.

In articles with data presented graphically, we contacted study authors via e-mail twice to request the numerical data. In case of no response, the data were extracted from the diagrams using WebPlotDigitizer 4.4. software, where possible.

### 2.6 Assessment of the risk of bias

The risk of bias was assessed with the Newcastle Ottawa scale (NOS) adapted for case–control studies, cross-sectional studies, and cohort studies (13). Risk of bias in included studies was assessed independently by two reviewers (IO and PK). In case of disagreement, the third investigator (MD) made the final decision.

NOS assesses bias in three domains: selection, comparability, and exposure for the case–control studies or outcome for the cohort studies. A star system is used in this scale; the scores on this scale range from 0 to 9 stars. Studies were rated based on scores from three domains and were given the final rate according to agency for healthcare research and quality (AHRQ) standards (good, fair, poor).

When investigating the expression of TSLP in asthma, the crucial point in outcome or exposure was the description of the TSLP detection method used, including the sensitivity threshold and/or detection range for TSLP in studies using ELISA and sequence of primers for TSLP in the PCR method.

### 2.7 Statistical analysis

All data were analyzed using Stata 18.0 software. A meta-analysis was performed when at least two studies measured TSLP expression in the same biological specimen using the same laboratory technique and reported results as mean with standard deviation.

Although seven studies assessed TSLP expression in bronchial biopsies, they were not included in the meta-analysis due to methodological heterogeneity. The studies assessed different anatomical sites within the bronchi (e.g., epithelium and submucosa), used different staining techniques and scoring methods, and reported results in non-uniform measurement units.

A random effect model was selected due to variability in laboratory techniques used. Outcomes were reported as mean and standard deviation. Effect sizes were pooled as standardized mean difference (SMD). Heterogeneity was used using *I*^2^, *H*^2^, and *τ*^2^ statistics. Heterogeneity was categorized as low (<25%), moderate (25%–50%), and high (>50%). A leave-one-out sensitivity analysis was conducted to assess the influence of individual studies on the overall effect estimate. Publication bias was assessed using Egger's test and funnel plots. To examine potential sources of heterogeneity in the meta-analysis, subgroup analysis based on the age of groups was conducted.

## 3 Results

### 3.1 Study selection

**Figure 1:**
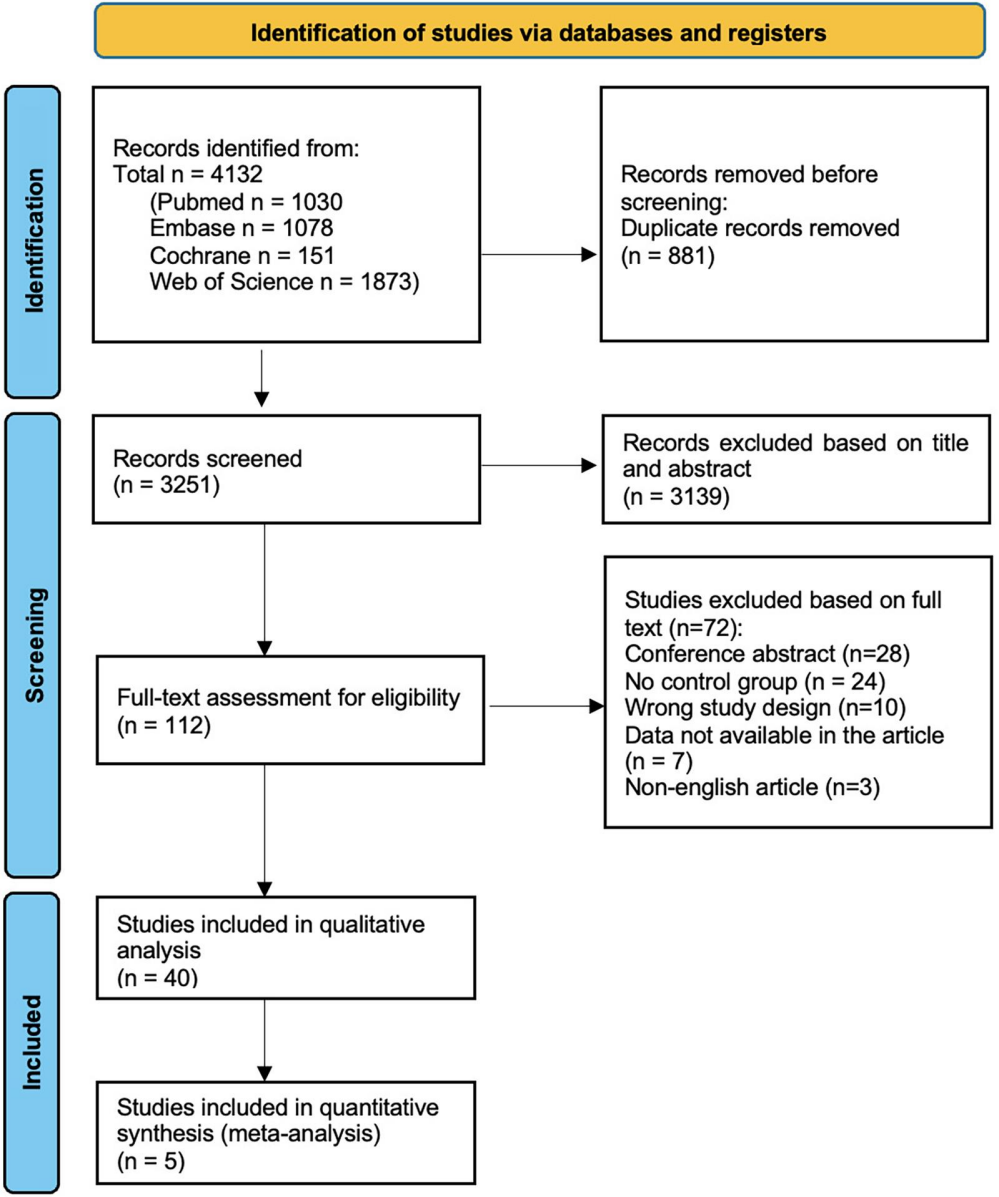
Prisma flow-chart of included studies in the systematic review and meta-analysis. Flow chart includes initial search and search-update.

A total of 4,132 citations were retrieved from the initial and update search of electronic databases. After removing 881 duplicates and 3,139 articles based on title and abstract, 112 articles remained for full-text review. Forty studies met all inclusion criteria and underwent data extraction and synthesis. The PRISMA flowchart is presented in Figure 1.

Six studies were eligible for the meta-analysis due to several factors. TSLP was measured in a range of airway specimens [e.g., bronchial biopsy, EBC, induced sputum, nasal lavage fluid, and nasal epithelial cells (NECs)], limiting direct comparisons between samples. Studies used different techniques to analyze TSLP concentrations, including ELISA, Meso Scale Discovery assay, and real-time PCR (RT-PCR).

Thus, only six studies measuring TSLP expression in the comparable specimens (blood including serum and plasma), using the same detection method (ELISA) and reporting results as mean and SD, were eligible for quantitative analysis. Of six studies eligible for meta-analysis, five had a case–control study design, and one was a cross-sectional study. One cross-sectional study was excluded from quantitative synthesis due to methodological heterogeneity. Finally, five case–control studies assessing TSLP concentration in blood were included in the meta-analysis. A narrative synthesis was performed for the remaining studies.

### 3.2 Basic characteristics of included studies

Details of the 40 included studies are presented in Table 1. Among these, 31 were case–control, 6 were cross-sectional, and 3 were cohort. The studies originated from four continents: 16 from Europe, 16 from Asia, 7 from North America, and 1 from Africa. Publication dates ranged from 2005 to October 2024.

**Table 1:** Characteristics of individual studies included in the meta-analysis.

	Study ID	Study design	Years	Country	Sample size		Mean age		Patients’ characteristics	Airway specimen	Method of TSLP detection	TSLP concentration		*p*-value
					Asthma	Controls	Asthma	Controls				Asthma	Controls	
1.	Ying et al. (36)	Case–control	2005	UK	20	15	Mild 33.5 (28–73)	36 (19–45)	6 mild, 7 moderate, 7 severe asthma	Bronchial biopsy—epithelium and submucosa	ISH	ND	ND	*p* < .05
							Moderate 45 (27–57)							
							Severe 45 (36–73)							
2.	Ying et al. (37)	Case–control	2008	UK	13	30	55 (31–73)	Never smoker 53 (41–68)	Moderate and severe asthma	Bronchial biopsy—epithelium and submucosa	ISH	ND	ND	*p* < .05
								Smoker 54 (32–71)		BALf	ELISA	ND	ND	*p* < .05
								Ex-smoker 61 (55–67)						
3.	Versluis et al. (43)	Case–control	2008	The Netherlands	17	10	32.29 ± 13.53	29.4 ± 7.34	ND	IS	ELISA	ND	ND	NS
4.	Semlali et al. (38)	Case–control	2010	Canada	10	10	24.7 ± 5.07	24.1 ± 4.30	Stable, mild asthma, no ICS,	Bronchial biopsy—cell culture supernatants	ELISA	76.9 ± 3.4 pg/mL	43.6 ± 2.9 pg/mL	*p* < .05
										Bronchial biopsy—baseline TSLP expression in epithelial cells	RT-PCR	4.98 ± 0.87^a^	1.00 ± 0.64^a^	*p* < .05
5.	Nguyen et al. (41)	Case–control	2010	USA	30 (AA 15, NA 15)	15	27.69 ± 8.16	All >17	ND	BALf	ELISA	ND	ND	*p* < .01 (AA vs. NA and vs. controls)
6.	Shikotra et al. (40)	Case–control	2011	UK	27	11	ND	ND	All severities	Bronchial biopsy—epithelium	Immunostaining	ND	ND	*p* < .05
									Moderate-to-severe asthma	Bronchial biopsy—lamina propria	Immunostaining	ND	ND	*p* < .05
										bronchial biopsy	qPCR	ND	ND	ND
7.	Kaur et al. (39)	Cross-sectional	2012	UK	30	9	Mild–moderate 53 (29) severe 51 (14)^c^	45 (27)	18 mild and moderate,	Bronchial biopsy—epithelium	Immunostaining—SQS	Mild–moderate 0.75 (1.9) ^a^	0 (0.3)^b^	*p* < .05 compared with the control group
									12 severe,	Bronchial biopsy—ASM	Immunostaining—SQS	Severe 0.75 (1.3)^b^	0.25 (0.4)^b^	*p* < .05
										Bronchial biopsy- lamina propria	Immunostaining	Mild–moderate 0.88 (0.7)^b^		Compared with the control group
										IS	ELISA	Severe 0.75 (0.9)^b^	2.2 (4.1) cells/mm^2^	*p* < .01
												Mild–moderate 16.3 (59.5)		Compared with the control group
												Severe 15.8 (17.7) cells/mm^2^		
												Below detection limit in 14/16 samples		-
8.	Koussih et al. (22)	Case–control	2012	Canada	48	15	All children 10–11 years old	-	35 allergic asthma, 13 non-allergic asthma	Serum	ELISA	ND	ND	NS
9.	Wu et al. (31)	Case–control	2012	China	10	10	29.7 ± 4.14	27.2 ± 5.31	ND	Bronchial biopsy—epithelium	Densitometry	2,224.82 ± 819.68	18,501.18 ± 936.76	*p* < .01
10.	Wu et al. (30)	Case–control	2013	China	10	10	29.7 ± 4.14	27.2 ± 5.31	ND	Bronchial biopsy—epithelium	Immunostaining (bimodal H score distribution)	26, 54 ± 2,65	8, 96 ± 1,02	*p* < .05
11.	Cheng et al. (15)	Case–control	2014	China	43	21	ND	35 ± 10	Newly diagnosed asthma	Bronchial brushings	qPCR	ND	ND	NS
12.	Manthei et al. (48)	Cohort study	2014	USA	46	13	ND	ND	Mild-to-moderate asthma,	Nasal lavage fluid	ELISA	Below the detection limit in all samples		-
13.	Bleck et al. (44)	Case–control	2015	USA	13	11	34 ± 2	33 ± 8	Mild asthma,	sHBECs	qPCR	1.1 × 10^−2^ (IQR, 0.8–1.9 pg/mL	0.5 × 10^−2^ (IQR 0.4–0.7 pg/mL	*p* = .001
14.	Chauhan et al. (16)	Case–control	2015	India	65	15	6.4 ± 3.2	8.0 ± 2.6	Newly diagnosed asthma	Plasma	ELISA	592 ± 68 pg/mL	215 ± 45 pg/mL	*p* = .041
15.	Han et al. (67)	Case–control	2016	China	20	20	12.0 ± 4.3	12.8 ± 4.9	ND	Blood	ELISA	387.9 ± 53.5 pg/mL	115.6 ± 30.8 pg/mL	*p* < .001
16.	Lai et al. (32)	Case–control	2016	China	44	22	44.5 ± 2.3 (SEM)	38.6 ± 1.8 (SEM)	8 mild asthma, 14 moderate, 22 severe,	Serum	ELISA	98.73 (61.57, 120.49) pg/mL	49.89 (27.07, 85.99) pg/mL	*p* < .001
17.	Berraïes et al. (28)	Case–control	2016	Tunisia	40	22	8 (4–16)	6.5 (4–12)	20 mild, 20 moderate,	IS	ELISA	239.80 ± 131.32 pg/mL	46.32 ± 34.65 pg/mL	*p* < .001
											ELISA	Mild asthma 133.70 ± 44.48 pg/mL	–	*p* < .001 (mild vs. moderate)
											ELISA	Moderate asthma 345.90 ± 98.60 pg/mL	–	
										IS	RT-PCR	1.2 (0.9, 1.4)^b^	0.05 (0.0, 0.19)^b^	*p* < .001
										Serum	ELISA	106.15 ± 51.13 pg/mL	8.4 ± 10.87 pg/mL	*p* < .001
												Mild asthma 81.85 ± 16.95 pg/mL	–	*p* < .001 (mild vs. moderate)
												Moderate asthma 150.45 ± 30.7 pg/mL	–	
18.	Glück et al. (25)	Case–control	2017	Poland	44 (AA-22, NA-22)	19	43 (33–54)	44 (38–56)	30 controlled asthma, 14 uncontrolled asthma,	Serum	ELISA	77 (42–129.1) pg/mL	45.9 (34.4–65) pg/mL	NS
												Controlled asthma (*n* = 30) 66.7 (40–124.9) pg/mL	Uncontrolled asthma (*n* = 14) 100 (54–144) pg/mL	NS (controlled vs. uncontrolled)
										EBC	ELISA	39 (23–55) pg/mL	13.1 (10.25–23.45) pg/mL	*p* = 0.0002
												Controlled asthma (*n* = 30) 41 (24.8–55)	Uncontrolled asthma (*n* = 14) 31 (12.5–40)	NS (controlled vs. uncontrolled)
19.	Górska et al. (35)	Cross-sectional	2016	Poland	24	12	51 (31–61)	55 (36–64)	Mild-to-moderate asthma	Serum	ELISA	36.5 (32.6–61.1) pg/mL	65.9 (44.0–90.7) pg/mL	NS
											ELISA	740 (560–835) pg/mL	895 (625–1,380) pg/mL	NS
										IS	ELISA	50.9 (34.2–73.2) pg/mL	12.2 (7.4–19.2) pg/mL	*p* = .05
											ELISA	50.7 (0–855) pg/mL	550 (325–728) pg/mL	*p* = .02
										EBC	ELISA	0 (0–0) pg/mL	0 (0–0) pg/mL	NS
											ELISA	0 (0.0–41.7) pg/mL	40 (0.0–76.7) pg/mL	NS
20.	Lin et al. (26)	Case–control	2016	China	31	53	7.84 ± 0.42	7.84 ± 0.43	Acute exacerbation; allergic asthma - 21, non-allergic asthma - 10	Serum	ELISA	273.00 ± 91.25 pg/mL	69.96 ± 15.21 pg/mL	*p* < .05
21.	Chai et al. (23)	Case–control	2017	China	64	32	31.9 ± 15	ND	allergic asthma - 32, non-allergic asthma - 32	Serum	ELISA	ND	ND	*p* < .05
											RT-PCR	ND	ND	*p* < .05
22.	Wang et al. (50)	Case–control	2018	China	124	50	48.49 ± 12.25	49.60 ± 18.14	ND	Plasma	ELISA	ND	ND	*p* < .001
23.	Li et al. (19)	Case–control	2018	China	70	30	42 (21–73)	26 (19–68)	29 steroid free, 21 ICS only, 20 ICS + OCS,	BALf	ELISA	ND	ND	*p* = .009
24.	Liu et al. (21)	Case–control	2018	USA	50	21	53.1 ± 4	54.3 ± 4	Moderate and severe, allergic asthma	BALf	ELISA	47.11 ± 17.17 pg/mL	22.35 ± 13.37 pg/mL	*p* = .002
25.	Kalinauskaite-Zukauske et al. (17)	Case–control	2019	Lithuania	9	9	25 ± 2.1	30 ± 2.9	Newly diagnosed, untreated, allergic asthma	Serum	ELISA	311.7 (216.9-581.7) pg/mL	319.1 (179.7-435.4) pg/mL	NS
26.	Ma et al. (20)	Case–control	2019	China	192	130	SSA 6.95 ± 1.72 SRA 7.01 ± 1.80	6.78 ± 1.73	SSA 110, SRA 82	Serum	ELISA	SSA 223.2 ± 24.9 pg/mL	89.1 ± 12.1 pg/mL	*p* < .05 (SSA vs. controls)
												SRA 244.5 ± 33.6 pg/mL		*p* < .05 (SSA vs. SRA)
27.	Majewski et al. (49)	Cross-sectional	2019	Poland	22	15	55.3 ± 3.0	57.9 ± 2.6	ND	EBC	ELISA	Undetectable	Undetectable	-
28.	Nejman-Gryz et al. (24)	Cross-sectional	2020	Poland	12	10	43.5 (27–59)	40.5 (29–63)	Allergic asthma	Serum	ELISA	7.3 (4.5–23.3) pg/mL	4.9 (0–6.9) pg/mL	NS
										IS	ELISA	3.5 (3–12.3) pg/mL	0 (0–0) pg/mL	*p* = .01
											qPCR	3.1 (1–13.2)^b^	0.16 (0.1–0.24)^b^	*p* = .01
29.	Kozlova et al. (68)	Cross-sectional	2020	Russia	62 (SAFS 25, SAwFS 37)	16	SAFS 44.0 (33.0–61.0)	24.0 (22.0–40.0)	ND	Serum	ELISA	SAFS 15.0 (9.5–27.5) pg/mL	10.5 (8.5–31.0) pg/mL	NS (all patients vs. HC)
							SAwFS 53.0 (32.5–64.0)					SAwFS 19.0 (15.0–29.0) pg/mL		NS (SAFS vs. SAwFS)
30.	Kim et al. (42)	Case–control	2020	Korea	107 (IS in 37)	19	53.32 ± 17.13	38.16 ± 9.65	ND	IS	RT-PCR	242.28 SEM 26.46^c^	33.67 SEM-10.82^c^	*p* < .001
31.	Zhang et al. (18)	Case–control	2021	China	57	26	40.0 (32.3–49.5) T2 low	35.0 (28.8–42.3)	20 T2 low asthma, 37 T2 high,	BALf	ELISA	T2 low 20.97 (18.49–24.30) pg/mL	19.14 (17.85–21.29) pg/mL	*p* < .001 (HC vs. asthma)
							42.0 (31.5–50.0) T2 high					T2 high 25.81 (20.32–30.54) pg/mL		*p* < .001 (T2 low vs. T2 high)
32.	Alturaiki et al. (14)	Case–control	2022	Saudi Arabia	31	19	35.04	33.07	Mild, chronic, stable asthma	Plasma	ELISA	78 ± 68 pg/mL	40 ± 5 pg/mL	*p* < .02
33.	Basu et al. (29)	Case–control	2022	Denmark	24	169	8.8 (6.9, 12.3)	7.1 (7.0, 7.5)	ND	Serum	Meso Scale Discovery-based	2.28 (1.12 −5.27) pg/mL	1.20 (0.54–2.04) pg/mL	*p* < .05
34.	Murrison et al. (47)	Case–control	2022	USA	32	19	6–18 years	6–18 years	ND	Plasma	ELISA	44.79 (33.64–67.28) pg/mL	39.67 (25.41–58.32) pg/mL	NS
										NECs—sfTSLP	RT-PCR	505.75 (285.06–733.33)^b^	197.7 (137.93–393.1)^b^	*p* = .003
										NECs—lfTSLP	RT-PCR	1.5 (0.66 −2.78)^b^	0.25 (0.18–1.74)^b^	*p* = .009
35.	Türk et al. (34)	Cohort study	2022	Turkey	76	15	45.6 ± 11.1	ND	Severe asthma	Serum	ELISA	105.2 (0) pg/mL	80.4 (0–169) pg/mL	NS
36.	Vrsalović et al. (52)	Cohort study	2022	Croatia	207	100	6 (3–9)	8 (5–100)	Mild and moderate asthma	Serum	ELISA	486 (117–1,053) pg/mL	302.5 (114.5–639.5) pg/mL	*p* = .001
37.	Andreasson et al. (53)	Cross-sectional	2023	Denmark	182	47	41 (26–54)	28 (25–47)	All severities	Serum	Meso Scale Discovery	Geo. mean:1.45 (95% CI: 1.35–1.55) fg/mL	0.85 (95% CI: 0.66–1.08) fg/mL	*p* < .001
38.	Ibrahim et al. (51)	Cross-sectional	2023	France	383	586	39.0 ± 16.5	46.2 ± 16.0	Non-severe asthma	Plasma	Meso Scale Diagnostics	2.69 (4.07–5.13) pg/mL	3.98 (2.82–6.31) pg/mL	NS
39.	Doulatpanah et al. (27)	Case–control	2023	Turkey	20	20	8.6 (7–11)	9.1 (7–12)	Asthma in clinical remission	Serum	Magnetic bead-based immunoassay	0.645 (0.430–0.843) pg/mL	0.430 (0.340–0.990) pg/mL	*p* = .03
										Nasal fluid	Magnetic bead-based immunoassay	0.515 (0.400–2.113) pg/μL	0.545 (0.390–1.350) pg/μL	NS
										Nasal fluid	Meso Scale Discovery	Eosinophil high geo.mean: 1.60 (95% CI: 1.47–1.74) fg/mL	–	*p* = .02 (eosinophil high vs. low)
												Eosinophil-low geo.mean: 1.30 (95% CI: 1.17–1.43) fg/mL		
40.	Połomska et al. (33)	Cross-sectional	2024	Poland	52	26	11.2 ± 3.10	12.69 ± 2.60	Mild asthma	Serum	ELISA	13.35 ± 10.94 pg/mL	20.86 ± 10.87 pg/mL	*p* = .008

Results are reported as mean and standard deviation (mean ± SD), median and interquartile range [median (Q1, Q3)], or if stated otherwise. AHR, airway hyperresponsiveness; EBC, exhaled breath condensate; HC, healthy controls; IS, induced sputum; ISH, *in situ* hybridization mRNA; ND, no data; NECs, nasal epithelial cells; NS, not significant; SAFS, severe asthma with fungal sensitization; SAwFS, severe asthma without fungal sensitization; SEM, standard error of the mean; sHBECs, sputum-derived human bronchial epithelial cells; SSA, steroid-sensitive asthma; SRA, steroid-resistant asthma; SQS, semiquantitative intensity score.

a Fold induction of normal epithelial cell expression.

b Expression relative to B-actin.

c 2^−ΔCt^ normalized to GAP.

A total of 2,361 patients with asthma and 1,704 healthy controls were included for the data synthesis. Eleven studies enrolled only children (aged 1–18 years), while 29 studies included only adults (over 18 years). The control groups were composed of healthy individuals or non-atopic patients without any pulmonary disease.

Twelve studies enrolled patients with varying asthma severity, divided into mild, moderate, and severe cases, while two included individuals with all asthma severities. The study by Alturaiki et al. (14) included patients with chronic stable, mild asthma. Four studies included untreated patients with newly diagnosed asthma (15–18). The study by Li et al. (19) included patients with variable control of asthma and stratified them according to treatment intensity: untreated, treated with inhaled corticosteroids (ICS), and treated with both ICS and oral corticosteroids (OCS). The study by Ma et al. (20) included patients with steroid-sensitive asthma and steroid-resistant asthma based on the improvement in FEV1 respiratory parameter following 7 days of treatment. The study by Liu et al. (21) enrolled 50 asthmatics with moderate and severe asthma; 10 among them were treated with OCS, and 13 received monoclonal antibodies—omalizumab. Studies by Koussih et al. (22) and Chai et al. (23) enrolled allergic and non-allergic asthmatics, while the study by Nejman-Gryz et al. (24) included only allergic asthmatic patients. The study by Glück et al. (25) compared TSLP concentration in controlled and uncontrolled asthmatics to healthy controls. The study by Lin et al. (26) included only patients with acute asthma exacerbation. The study by Doulatpanah et al. (27) enrolled asthmatic children in clinical remission. A study Zhang et al. (18) enrolled 57 newly diagnosed, treatment-naive patients and divided the asthmatic study group into T2-high and T2-low endotypes based on the expression of certain genes in epithelial brushings. In 11 studies, the severity and clinical profile of the asthmatic population were not specified.

Twenty-three studies assessed TSLP expression in blood; specifically, 17 studies measured TSLP in serum, 5 in plasma, and 1 study in blood (not specified). Thirteen studies assessed TSLP in airway-derived specimens, including seven in bronchial biopsies, five in BALf, and one in bronchial brushings. Seven studies analyzed induced sputum: six measured TSLP expression in sputum samples and one measured it in sputum-derived human bronchial epithelial cells (sHBECs). Three studies used nasal specimens: two analyzed nasal lavage fluid and one analyzed nasal epithelial cells (NECs). In addition, three studies measured TSLP in exhaled breath condensate (EBC). Several studies analyzed TSLP expression in more than one biological specimen, and some measured TSLP expression in the same biological specimen using two laboratory methods (ELISA and RT-PCR). Details of ELISA and other assays used in studies, including manufacturers, sensitivity, detection thresholds, and detection ranges, are presented in Supplementary Table S1.

Sixteen studies reported results as numerical values; nine studies reported TSLP expression levels as median with interquartile range (IQR), whereas seven articles reported TSLP as means with standard deviation (SD). Twenty-two studies reported results on the graphs, without providing numerical values. In the two included articles, TSLP in airway specimens was below the detection limit.

Of the 40 included studies, 29 reported funding support from grants or institutions. Four studies declared no funding, and funding information in seven studies was not reported. Details of funding sources and potential conflicts of interest are provided in Supplementary Table S2.

Results of TSLP expression levels in asthmatics and healthy controls were obtained directly from 21 articles, while data from 8 studies were extracted using Digitizer software (18, 21, 26, 28–32). In 10 studies, graphs were ineligible for numerical data extraction using this software.

### 3.3 Risk of bias

The quality rating was qualified as good in 27 studies and fair in 10 studies, overall indicating low risk of bias. Two studies were rated as low quality. In the study by Połomska et al. (33), the group of asthmatic children and healthy controls were not comparable; the group of healthy controls had significantly higher BMI and was older compared with asthmatics. The study by Türk et al. (34) was underpowered to detect significant differences between the two groups due to an insufficient study sample. The outcome of the quality rating of each domain is summarized in Table 2.

**Table 2:** Quality rating of included studies in the systematic review and meta-analysis.

Study ID	Study design	Selection	Comparability	Outcome/exposure	Total score	Quality rating
Ying et al. (36)	Case–control	★★★	★★	★★★	8	Good quality
Ying et al. (37)	Case–control	★★★	★★	★★★	8	Good quality
Versluis et al. (43)	Case–control	★★★★	★	★★	7	Good quality
Semlali et al. (38)	Case–control	★★	★★	★★★	6	Good quality
Nguyen et al. (41)	Case–control	★★★★	★★	★★	8	Good quality
Shikotra et al. (40)	Case–control	★★★	★★	★★★	8	Good quality
Kaur et al. (39)	Cross-sectional	★★	★	★★	5	Fair quality
Koussih et al. (22)	Case–control	★★	★	★★★	6	Fair quality
Wu et al. (31)	Case–control	★★	★★	★★	6	Fair quality
Wu et al. (30)	Case–control	★★	★★	★★	6	Fair quality
Cheng et al. (15)	Case–control	★★	★★	★★	6	Fair quality
Manthei et al. (48)	Cohort study	★★	★★	★★	6	Fair quality
Bleck et al. (44)	Case–control	★★★	★★	★★★	8	Good quality
Chauhan et al. (16)	Case–control	★★★★	★	★★	7	Good quality
Han et al. (67)	Case–control	★★★★	★★	★★	8	Good quality
Lai et al. (32)	Case–control	★★★	★★	★★★	8	Good quality
Berraïes et al. (28)	Case–control	★★★	★★	★★★	8	Good quality
Glück et al. (25)	Case–control	★★★	★★	★★	7	Good quality
Górska et al. (35)	Cross-sectional	★★★★	★★	★★★	9	Good quality
Lin et al. (26)	Case–control	★★★	★★	★★	7	Good quality
Chai et al. (23)	Case–control	★★★	★★	★★	7	Good quality
Wang et al. (50)	Case–control	★★★★	★	★★	7	Good quality
Li et al. (19)	Case–control	★★★★	★★	★★★	9	Good quality
Liu et al. (21)	Case–control	★★★	★★	★★★	8	Good quality
Kalinauskaite-Zukauske et al. (17)	Case–control	★★★★	★★	★★★	9	Good quality
Ma et al. (20)	Case–control	★★★★	★★	★★	8	Good quality
Majewski et al. (49)	Cross-sectional	★★	★★	★★	6	Fair quality
Nejman-Gryz et al. (24)	Cross-sectional	★★★★	★★	★★★	9	Good quality
Kozlova et al. (68)	Cross-sectional	★★	★★	★★	6	Fair quality
Kim et al. (42)	Case–control	★★★	★	★★	6	Good quality
Zhang et al. (18)	Case–control	★★★	★★	★★	7	Good quality
Alturaiki et al. (14)	Case–control	★★★	★	★★	6	Good quality
Basu et al. (29)	Case–control	★★★★	★★	★★★	9	Good quality
Murrison et al. (47)	Case–control	★★★★	★★	★★★	9	Good quality
Türk et al. (34)	Cohort study	★★	★★	★	5	Poor quality
Vrsalović et al. (52)	Cohort study	★★★★	★★	★★	8	Good quality
Andreasson et al. (53)	Cross-sectional	★★★★	★	★★★	8	Fair quality
Ibrahim et al. (51)	Cross-sectional	★★	★★	★★★	7	Fair quality
Doulatpanah et al. (27)	Case–control	★★★	★★	★★	7	Good quality
Połomska et al. (33)	Cross-sectional	★★★★	-	★★	6	Poor quality

In the selection domain, the maximum score is four stars; in the comparability domain, two stars; and in outcome or exposure, three stars.

### 3.4 Meta-analysis

#### 3.4.1 Blood TSLP concentrations in asthmatic patients and healthy controls

**Figure 2:**
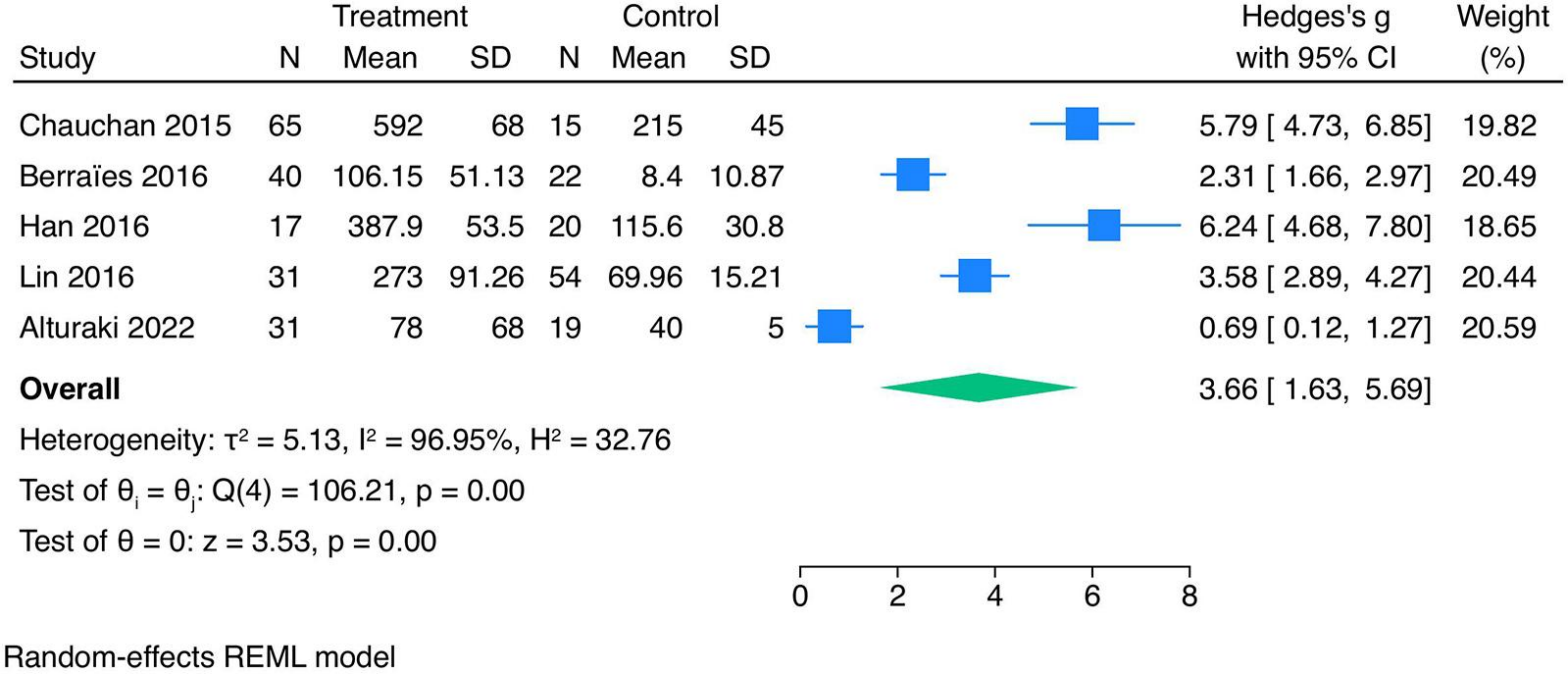
Meta-analysis of the studies analyzing TSLP expression in blood in the group of asthmatics and healthy controls.

In the meta-analysis of five included studies, TSLP concentration levels were significantly higher in the group of asthmatics than in the control group (SMD = 3.66, 95% CI 1.63–5.69) (Figure 2).

**Figure 3:**
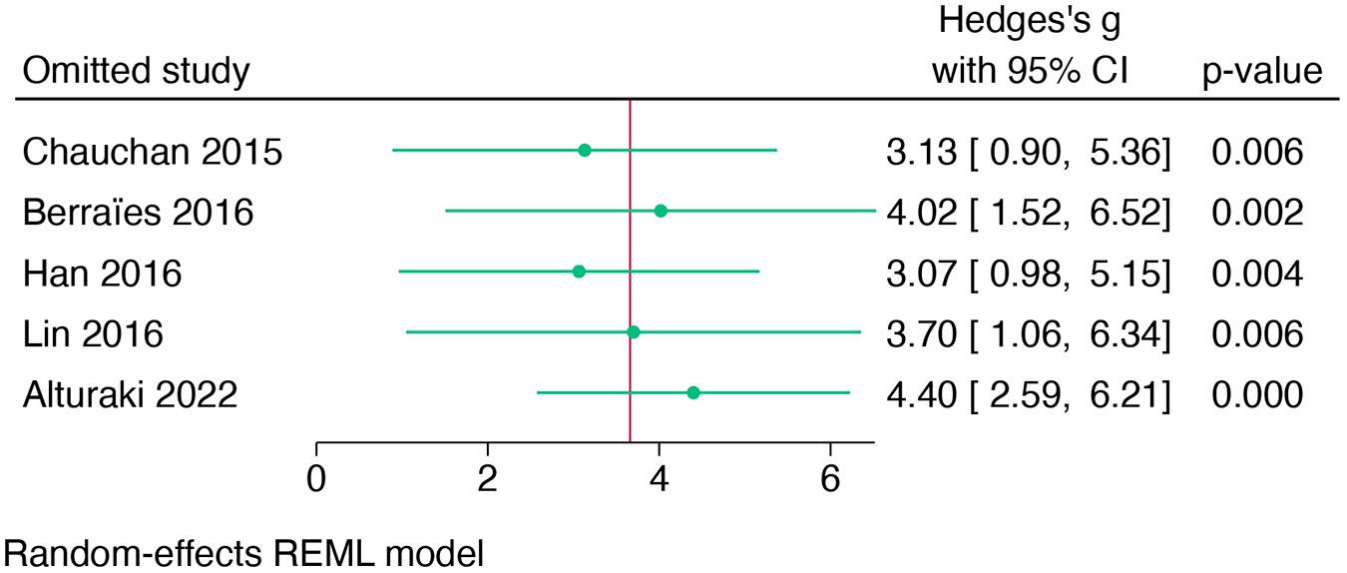
Leave-one-out sensitivity analysis of studies comparing TSLP expression in blood between patients with asthma and healthy controls.

The total heterogeneity was very high (*I*^2^ = 98.26%). However, the sensitivity analysis (leave-one-out) showed that no individual study influenced the pooled effect estimate, confirming the robustness of the findings (Figure 3). Meta-regression was not conducted due to the limited number (*n* = 5) of studies in the meta-analysis.

**Figure 4:**
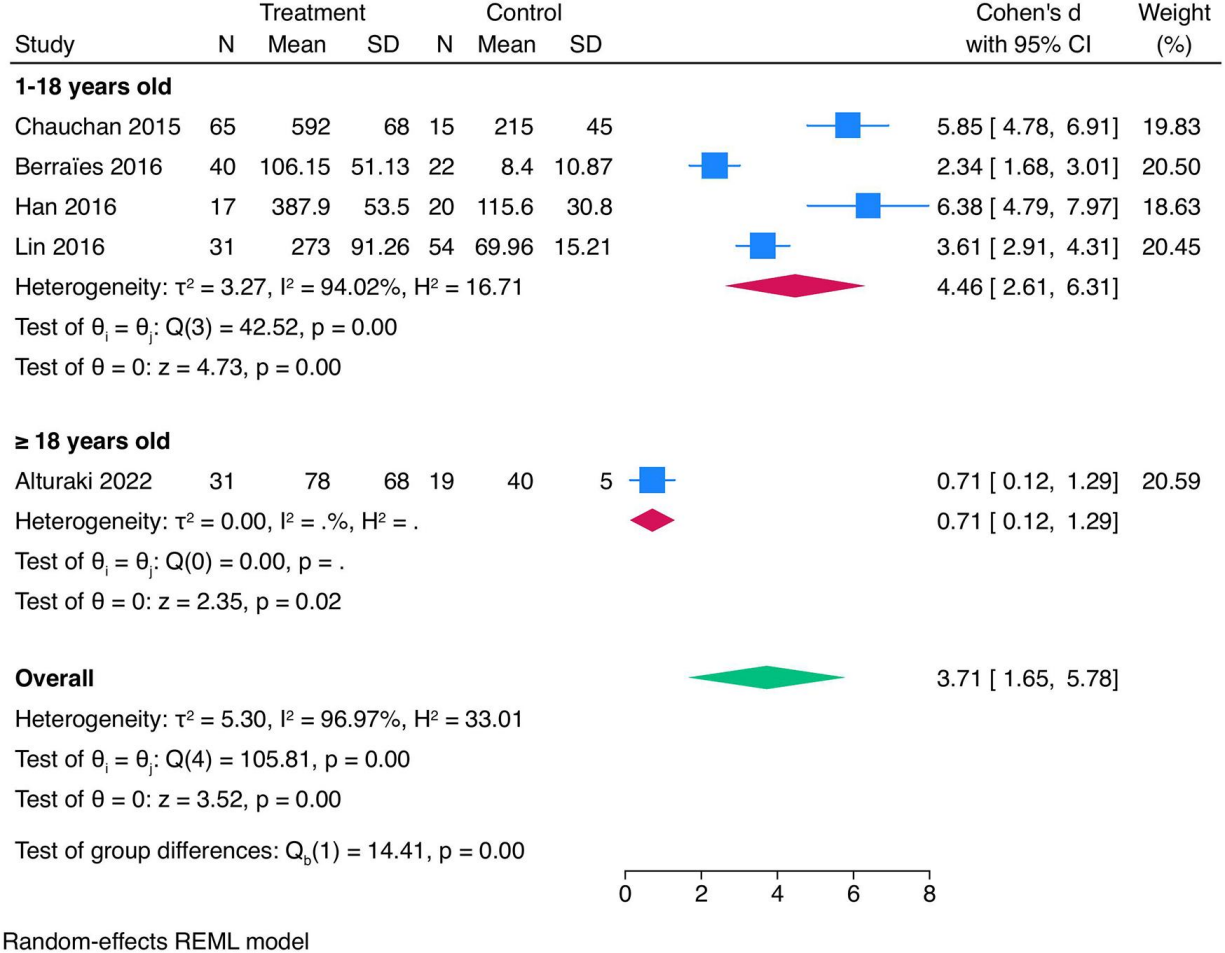
Subgroup meta-analysis bases on age. Forest plot shows pooled differences in TSLP concentrations between healthy controls and asthmatic patients stratified into children (1–18 years old) and adults (over 18 years old).

A subgroup analysis based on age was conducted to explore potential sources of heterogeneity (Figure 4). In the subgroup analysis, children (1–18 years old) showed higher serum concentrations of TSLP compared with the adult population (≥18 years old). The pooled SMD for children (1–18 years old) was 4.46 (95% CI 2.61–6.31) while SMD in the single study of the adult population was 0.71 (95% CI 0.12–1.29). The test for subgroup differences was statistically significant (Qb = 14.41, *p* < 0.001), suggesting that age may account, at least in part, for the observed heterogeneity. However, these findings should be interpreted with caution, given the inclusion of only one study in the adult group.

#### 3.4.2 Publication bias

**Figure 5:**
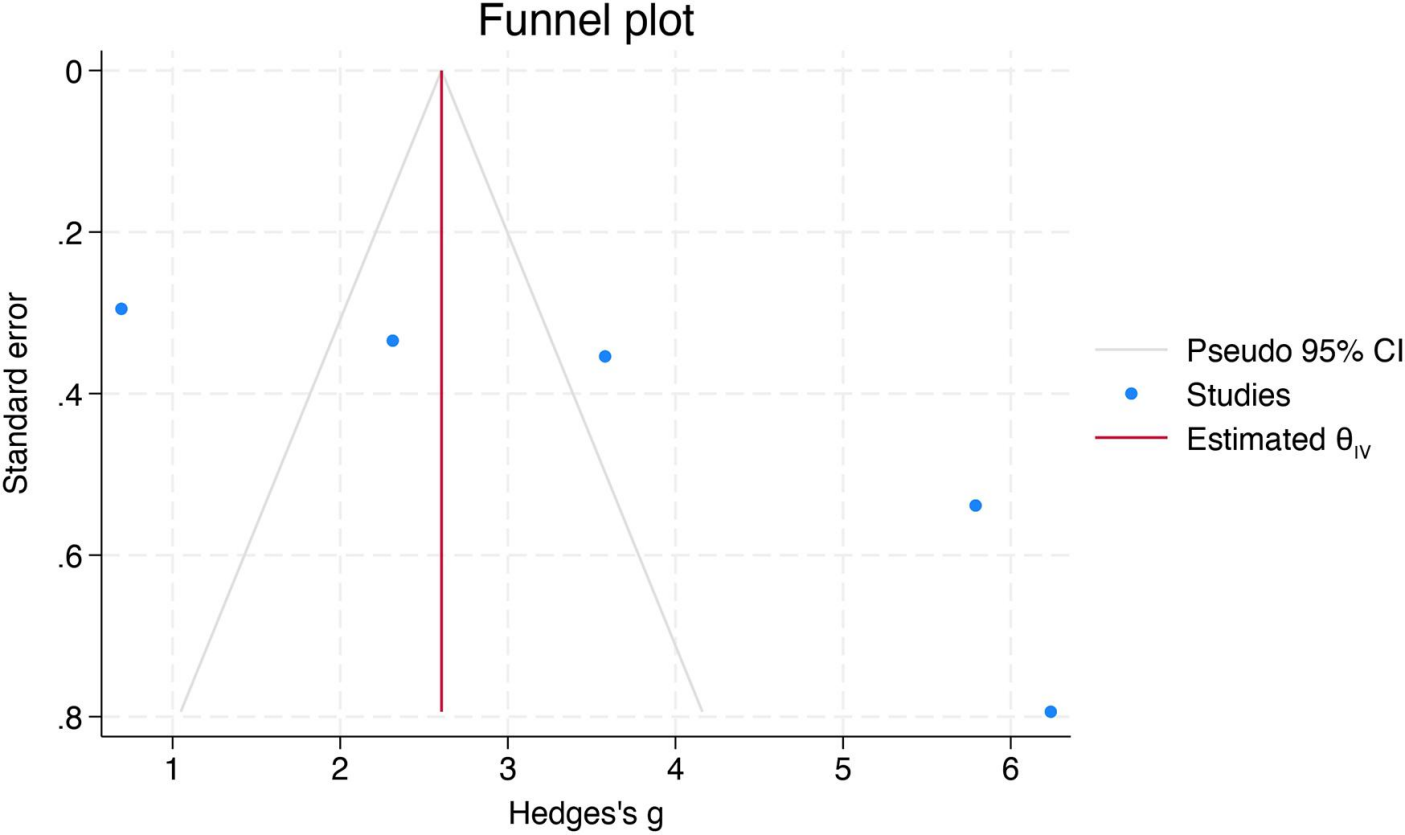
Funnel plot evaluating publication bias.

**Figure 6:**
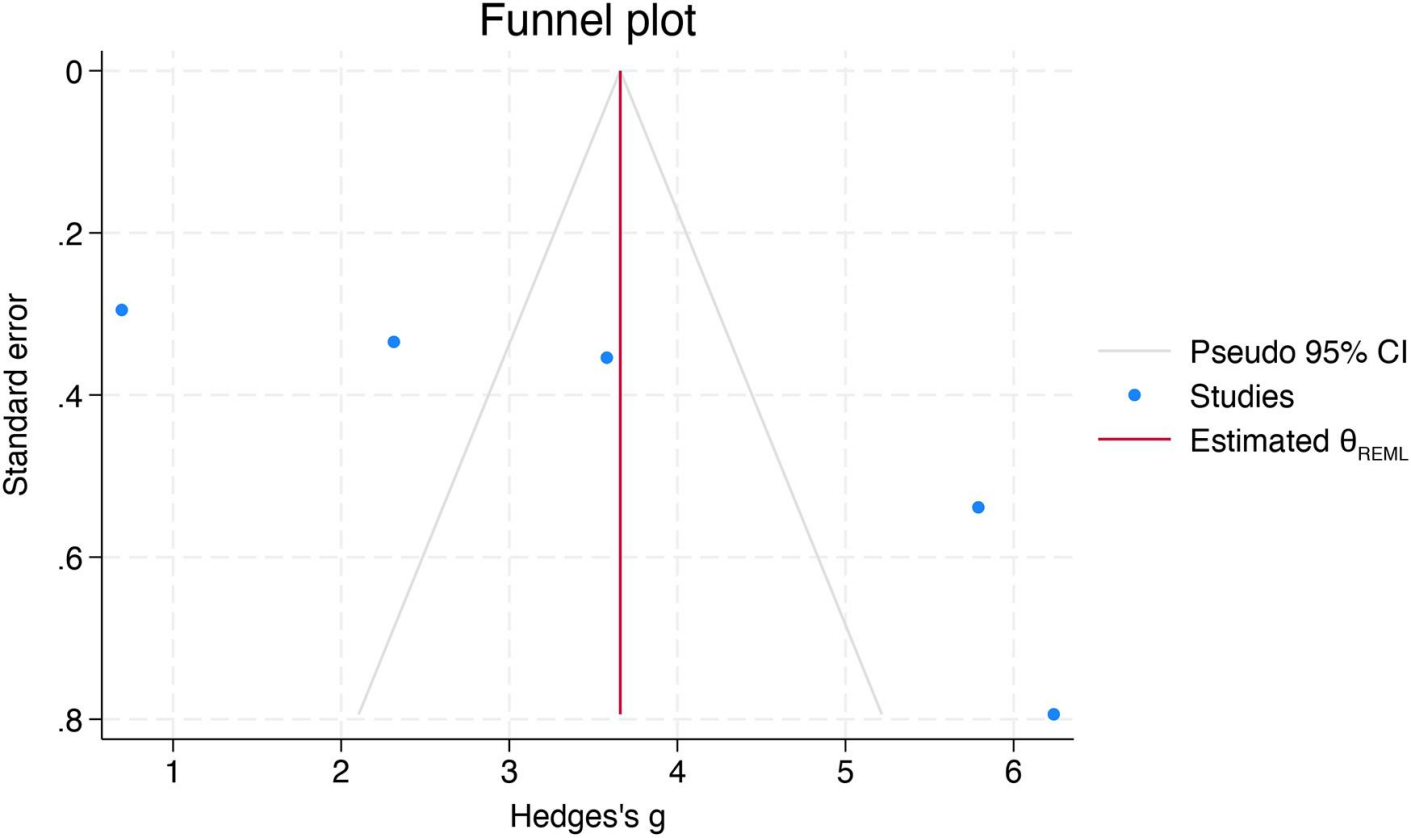
Funnel plot after applying the “trim-and-fill” method, illustrating the adjusted effect estimates.

The funnel plot showed moderate asymmetry (Figure 5), and the “trim-and-fill” method was used to adjust for bias. Although no missing studies were imputed, the pooled SMD was recalculated (Figure 6).

Egger's test indicated evidence of small-study effects, indicating potential publication bias (*p* = 0.002). Begg's test did not reach statistical significance (*p* = 0.09). Overall, these findings show small-study effects indicative of publication bias.

### 3.5 Systematic review

A total of 40 studies were included for the qualitative analysis, and 5 articles were included for quantitative synthesis.

#### 3.5.1 TSLP concentration in blood

Significantly higher blood TSLP in asthmatics than in healthy controls was reported in 15 out of 23 included articles. Only one study reported higher expression of TSLP in healthy controls compared with the asthmatic group (33). However, in this study, the healthy control group was significantly older and had higher BMI than the asthmatic group, potentially confounding the results of TSLP expression. In seven articles, the difference in blood TSLP concentration in the asthmatic and control groups was insignificant.

Specifically, 18 studies measured TSLP protein concentration in blood using ELISA, and 4 studies used Meso Scale Discovery. The study by Chai et al. (23) measured TSLP expression twice using the same blood specimens—expression of TSLP mRNA using RT-PCR and TSLP concentration using ELISA. The study by Górska et al. (35) measured TSLP in the same blood specimens using two ELISA kits (EIAab and R&D).

#### 3.5.2 TSLP expression in bronchial biopsies

Seven studies measured TSLP expression in bronchial biopsies; however, two articles analyzed the same study population. However, the staining technique was different, and both reports were included in further analysis.

All seven studies assessing TSLP expression in bronchial biopsies (including bronchial epithelial cells, lamina propria, and airway smooth muscles) reported a significant difference between asthmatics and healthy controls.

The study by Wu et al. (31) analyzed biopsy specimens (epithelium), and TSLP was quantified by densitometry. Another study by the same author analyzed expression of TSLP in the same subset of patients, using immunohistochemical staining and immunohistochemical scoring (bimodal *H* score distribution) in bronchial epithelium (30). Studies by Ying et al. (36, 37) identified TSLP mRNA-expressing cells in the bronchial epithelium and submucosa, using *in situ* hybridization (ISH). Semlali et al. (38) examined the baseline TSLP mRNA expression in epithelial cells obtained from bronchial biopsies using quantitative RT-PCR. TSLP protein was also assessed in cell culture supernatants from asthmatic and control subjects. In the study by Shikotra et al., TSLP expression was examined in bronchial biopsies (epithelium and lamina propria) by two methods; the first was based on the intensity of hue saturation staining, and the second method was based on a 4-point scale of semiquantitative scoring (28). Kaur et al. (39) analyzed expression of TSLP in the airway smooth muscle (ASM) and lamina propria using semiquantitative scoring, red hue staining, and cells expressing TSLP per square millimeter in lamina propria, ASM, and epithelium.

In two studies, biopsies were obtained from the middle and lower bronchi of the right lobe (36, 40). In the study by Ying et al. (37), biopsies were taken from the lower and segmental bronchi of the right lower, middle, and upper lobes, all performed by a single operator. In the four remaining articles, the bronchoscopy technique and anatomical sites of biopsy were not detailed (30, 31, 38, 39).

Due to heterogeneity in outcome reporting and analysis of different regions within bronchial biopsy specimens, a meta-analysis of these studies could not be conducted.

#### 3.5.3 TSLP concentration in BALf and bronchial brushings

Of the five studies analyzing TSLP concentration in BALf (18, 19, 21, 37, 41) and one measuring TSLP in bronchial brushings (15), all reported significantly higher TSLP levels in asthmatics compared with healthy controls. Studies used different volumes of sterile saline for BALf collection; Li et al. (19) used 4 × 60 mL, Liu et al.—2 × 60 mL (21), while Zhang et al. (18) used 40 mL of sterile saline. None of the studies reported sufficient data to allow correction for dilutional factors or normalization (e.g., to urea concentration or total protein content). The study by Ying et al. (37) and Nguyen et al. (41) analyzed undiluted BALf samples.

#### 3.5.4 TSLP concentration in induced sputum

Six studies assessed TSLP concentration in induced sputum; four of them reported significantly higher levels in asthmatics compared with healthy controls (24, 28, 35, 42). In the study by Kaur et al. (39), TSLP was undetectable in 14 of 16 samples, although the sample handling procedures were not detailed, limiting interpretability. Versluis et al. (43) found no difference in TSLP expression in induced sputum between groups. Bleck et al. (44) assessed TSLP expression in sHBECs using RT-PCR and reported higher expression in asthmatics. Studies by Górska et al. (35) and Nejman-Gryz et al. (24) followed the European Respiratory Society (ERS) standardized methodology of sputum induction and processing (45). Two studies (28, 44) also adhered to a standardized method (46), while the remaining studies detailed sputum processing in the methods section of each article.

#### 3.5.5 TSLP concentration in nasal epithelial cells, nasal lavage fluid, and EBC

Murrison et al. (47) measured expression of two TSLP isoforms using RT-PCR in nasal epithelial cells; concentrations of lfTSLP and sfTSLP were significantly higher in asthmatics compared with healthy controls. Two studies measured TSLP expression in nasal lavage fluid; none of them found a significant difference between groups (27, 48). Three studies assessed TSLP in EBC; in the study by Górska et al. (35), TSLP was undetectable in most samples (in 100% and 54% of samples using R&D and EIAab ELISA kits, respectively). In the study by Majewski et al. (49), the TSLP protein was undetectable in all EBC samples. Glück et al. (25) reported higher TSLP concentrations in EBC of asthmatic subjects compared with healthy controls.

### 3.6 Additional outcomes

We reviewed included studies to identify any additional outcomes, including associations between TSLP concentrations in different biological specimens and factors such as asthma severity, asthma control, age, sex, ethnicity, lung function, total serum IgE concentration, and family history of asthma/allergy.

#### 3.6.1 Correlation between respiratory parameters and TSLP

Nine studies examined the correlation between TSLP concentration and lung function. Six studies reported significant inverse correlation: five studies between TSLP expression and spirometry parameters (FEV1%, FEV1/FVC, FVC%) (19, 28, 36, 40, 50, 51). Three studies found no correlation between TSLP and lung function parameters (22, 25, 52). The correlation coefficients, strengths of correlations, and type of analyzed biological specimens are summarized in Table 3.

**Table 3:** Summary of correlation of TSLP expression with lung function parameters in different airway specimens.

Study ID	Number of asthmatic patients	Method	Airway specimen and detection method	Lung function parameter	Correlation coefficient (*R*)	Strength of association
Ying et al. (36)	*n* = 13	Spearman rank-order method	Epithelial cells expressing TSLP mRNA (ISH)	FEV1	−0.675	Strong
			Submucosal cells expressing TSLP mRNA (ISH)	FEV1	−0.549	Moderate
Shikotra et al. (40)	*n* = 27	Spearman rank-order method	Bronchial lamina propria	FEV1/FVC	−0.530	Moderate
			Bronchial epithelium	FEV1/FVC	−0.400	Moderate
Berraïes et al. (28)	*n* = 40	Spearman rank-order method	TSLP mRNA in serum (RT-PCR)	FEV1%pred.	−0.736	Strong
Li et al. (19)	*n* = 70	Spearman rank-order method	BALf (ELISA)	FEV1%pred.	−0.565	Moderate
Wang et al. (50)	*n* = 124	Spearman rank-order method	Plasma (ELISA)	FEV1%pred.	−0.258	Very weak
			Plasma (ELISA)	FVC%pred.	−0.267	Very weak
			plasma (ELISA)	FEV1/FVC%	−0.174	Very weak
Ibrahim et al. (51)	*n* = 383	Pearson correlation	Plasma (Meso Scale Discovery)	FEV1%pred.	−0.23, 95% CI −0.32 to−0.13	Very weak
			Plasma (Meso Scale Discovery)	FEV1/FVC	−0.25, 95% CI −0.34 to −0.15	Very weak
			Plasma (Meso Scale Discovery)	FEF 25–75%pred.	−0.16 95% CI −0.26 to −0.06	Weak

Strength of correlation was classified as strong (− 0.71 to −0.90), moderate (−0.51 to −0.70), weak (−0.31 to −0.50), or very weak (−0.01 to −0.30).

#### 3.6.2 TSLP and asthma severity

Due to inconsistent reporting, variations in measurement methods, and heterogeneity of available data, additional results are presented in a narrative synthesis.

Berraïes et al. (28) showed that TSLP concentration in induced sputum and serum was significantly higher in moderate asthmatics compared with patients with mild asthma. In the study by Kaur et al. (39), TSLP expression in bronchial biopsies was higher in mild-to-moderate asthmatics compared with healthy controls; however, TSLP expression in severe asthmatics was not significantly elevated. In the study by Shikotra et al. (40), subgroup analysis revealed significantly higher TSLP expression in the epithelia of both mild and severe asthma compared with healthy controls with elevated TSLP expression observed across the spectrum of disease severity.

#### 3.6.3 Other outcomes

The study by Basu et al. (29) reported a positive weak correlation between participants' age and TSLP levels (*r* = 0.23, *p* < .001). A cross-sectional analysis by Ibrahim et al. (51) showed that TSLP plasma concentrations were associated with increased age, BMI, and male sex. In a report by Andreasson et al. (53), higher serum TSLP levels were linked with older age, male sex, and higher blood eosinophil count. TSLP concentrations in BAL and sputum increased with older age (>50 years) and higher BMI (>30) (only in BAL samples) (53). The study by Kaur et al. (39) reported no relationship between TSLP and adipokines such as leptin and adiponectin.

Two studies reported a weak negative correlation between TSLP concentration and asthma control measured by asthma control test (ACT) in the asthmatic group (16, 20). Two studies reported no association between any allergy and TSLP expression among asthmatic patients (14, 52). In the study by Zhang et al. (18), TSLP concentration in BALf and bronchial brushings was higher in the T2-high asthmatic group compared with the T2-low group. The study by Shikotra et al. (40) reported a correlation between expression of TSLP in lamina propria and expression of T2-related cytokines (*r* = 0.27, *p* = .049, *n* = 53). Weak correlation between lamina propria TSLP count and reliever use per week (*r* = 0.4, *p* = .045) was also reported (40).

## 4 Discussion

### 4.1 Main findings

Our meta-analysis of five studies suggests that blood TSLP in patients with asthma is higher compared with healthy controls; however, due to variability in patients' selection (as far as age or asthma control and severity are concerned), variability in methodology, and laboratory methods, these findings should be interpreted with caution. Based on a systematic review, 15 out of 23 studies assessing blood TSLP (serum or plasma) reported significantly higher TSLP concentration in asthmatic subjects compared with healthy controls. All seven studies measuring TSLP in bronchial biopsy specimens, and all six studies analyzing TSLP in BALf and bronchial brushings reported higher TSLP expression in patients with asthma compared with healthy controls. Results of TSLP concentration in other airway specimens (induced sputum, nasal epithelial cells, nasal lavage fluid, and EBC) were inconsistent.

Higher concentration of TSLP in blood and other airway specimens of asthmatic patients may be a premise to check if it could be a useful disease-related marker.

### 4.2 Interpretation of findings in the light of existing literature

A meta-analysis of five studies suggested higher blood TSLP in asthmatic patients compared with healthy controls. However, based on a systematic review of studies analyzing blood TSLP, the results were variable. All studies assessing TSLP in specimens from the lower respiratory tract (BALf and bronchial biopsy) included in the systematic review reported significantly higher TSLP expression in asthmatics compared with healthy controls.

Based on available data, it remains unclear whether circulating TSLP concentrations reflect local expression of TSLP in the lungs. Factors influencing the release of TSLP into circulation are unknown. Further research is needed to clarify this relationship and determine whether serum TSLP can serve as a reliable marker for airway inflammation in asthma.

Some studies reported a lack of correlation between serum levels of TSLP and its expression in airway specimens, such as nasal epithelial cells (47) and exhaled breath condensate (25), but this may result from variable results of studies on TSLP in EBC or nasal specimens. One study reported a positive correlation between TSLP concentrations in blood and in induced sputum (53). Evidence on the correlation between circulating TSLP and blood eosinophil count is inconsistent; one study reported an association between blood TSLP and blood eosinophil count (53), while others reported no correlation (54).

### 4.3 Factors influencing TSLP expression in the lungs

Multiple factors impact TSLP expression such as environmental exposures (allergens, respiratory infections, air pollution) and host factors (age, gender, BMI, genetic polymorphisms) (5). Exposure to air pollutants, such as diesel exhaust particles (DES), a component of particulate matter (PM), stimulates the airway epithelium to release TSLP and initiate the airway T2 inflammatory response (55). Certain single-nucleotide polymorphisms (SNPs) in the TSLP locus have been linked to increased asthma susceptibility and upregulated TSLP expression (56). TSLP polymorphism in the rs1837253 locus may be directly involved in the regulation of TSLP secretion (57). Higher blood TSLP levels are associated with male sex (51), higher BMI (33), older age (29, 51), and smoking status (37, 51). In humans, TSLP is expressed by differentiated adipocytes, and serum TSLP concentration is related to basal metabolic index (58, 59). TSLP released from sources other than the lungs—such as adipose tissue—might potentially affect airway inflammation and influence asthma severity, partially explaining higher blood TSLP concentrations in patients with higher BMI. Based on current evidence, clinical atopy might not affect TSLP expression (14, 52). However, a significant increase in blood TSLP concentration was reported in asthmatics following allergen challenge with house dust mite (17, 43, 60).

Another factor influencing TSLP levels is treatment with inhaled and systemic corticosteroids. TSLP has been demonstrated in both murine and human models to promote corticosteroid resistance in group 2 innate lymphoid cells (ILC2) by activation of the STAT5 signaling pathway (21, 61). Conversely, blockage of the TSLP signaling pathway might restore corticosteroid resistance and improve the response of ILC2s to corticosteroids (21, 61). TSLP serum levels were higher in asthmatic children with steroid resistance (defined as <10% improvement in FEV1% following 7-day treatment with budesonide, 400 μg/day, and β2 receptor agonist 200 μg/day) compared with steroid-sensitive patients (with >10% improvement in FEV1%) (20). In the group of patients with asthma exacerbation, TSLP levels decreased following OCS treatment in the steroid responders' group; however, in the group of paradoxical responders, TSLP levels did not decrease after OCS treatment during asthma exacerbation (62). Semlali et al. (38) analyzed TSLP receptor (TSLPR) expression in bronchial biopsies before and after an 8-week course of ICS and reported no significant change in TSLPR immunostaining scores following treatment. Thus, TSLP concentrations might be elevated in corticosteroid-resistant asthmatic patients despite ICS or OCS treatment.

### 4.4 Strong points

Our study holds notable strengths as the first systematic review and meta-analysis of the concentration and expression of TSLP in blood and airway specimens of asthmatic patients. We conducted a comprehensive analysis of forty studies assessing TSLP concentrations in different biological specimens in asthmatic patients compared with healthy controls.

Based on a meta-analysis of five eligible studies, TSLP concentration in blood might be elevated in asthmatic patients compared with healthy controls. However, further studies are needed to validate this finding. Based on the systematic review, TSLP expression was consistently upregulated in specimens from the lower respiratory tract—bronchial biopsies and BALf.

### 4.5 Limitations of the study

A major limitation of our study is the inconsistency in reporting TSLP concentrations, with data presented as either median and IQR or mean and SD, complicating data synthesis for meta-analysis. Laboratory measurements including TSLP concentrations in biological specimens are commonly non-normally distributed and mostly reported as median and IQR (47). In one included study (53), the distribution of serum TSLP concentrations in patients with asthma (*n* = 182) and healthy controls (*n* = 47) demonstrated a non-normal, right-skewed pattern in both groups. Moreover, TSLP concentrations in induced sputum (*n* = 71) and bronchoalveolar lavage fluid (*n* = 85) from patients with asthma also exhibited non-normal, right-skewed distributions. Among the 40 studies included in the systematic review, only 7 studies reported TSLP concentration as mean and SD.

Although mathematical methods to convert median and IQR to mean and SD exist, they may introduce significant bias (63). We decided to include in the meta-analysis only studies reporting TSLP concentrations as mean and SD. Secondly, in some articles, data were only available on the graphs, with raw numerical values unavailable. Despite attempts to contact authors, responses were incomplete with limited data retrieval.

Another limitation of our meta-analysis is that four out of five eligible studies enrolled children (1–18 years old), limiting the reliability and applicability of findings to the general population. In addition, asthmatic populations varied across included studies in terms of asthma phenotypes (e.g., atopic vs. non-atopic), disease severity (mild vs. severe asthma), and age (children vs. adults), potentially influencing TSLP levels. The reproducibility of TSLP measurements could also influence the comparability between studies, given that TSLP is typically present at low concentrations and has a short half-life. The precision of TSLP measurements may be influenced by both biological variability (e.g., diurnal fluctuations, immune activation, and responses to exogenous factors such as allergens or air pollution) and technical variability (e.g., pre-analytical processing, non-standardized sample collection methods, and differences in assay sensitivity and detection thresholds).

High heterogeneity of studies is due to different measurement techniques including ELISA, PCR, immunostaining, and Meso Scale Discovery assays. Although ELISA was the most used method, studies used kits from various manufacturers, each with different detection thresholds and sensitivity levels (Supplementary Table S1). Górska et al. (35) assessed TSLP expression in the same biological samples from the same study population using two different ELISA kits (EIAab kit, R&D kit). TSLP concentration levels measured with the EIAab kit were between 3- and 45-fold higher than those obtained using the R&D kit (35). Thus, comparing TSLP expression levels across studies using ELISA kits from different manufacturers, with different sensitivity and detection thresholds, may be biased. Moreover, the choice of either plasma or serum may also be an issue in the measurement of biomarkers. Although we did not find any study on the direct comparison of serum and plasma TSLP concentration, we assume that, similar to the other methods, these values are not identical, but have linear serum-plasma relationships (64). Such assumptions allow us to hypothesize that the difference between patients with asthma and controls) may be identified independently of the choice of specimen.

In several biological specimens such as EBC and induced sputum, TSLP was undetectable, which may be due to either insufficient sensitivity of the ELISA kits used or inherently trace amounts of TSLP in certain biological specimens. Another potential confounder may arise from inappropriate sample collection, pre-analytical processing, and laboratory handling of biological specimens. In one study, TSLP was undetectable in 14 of 16 sputum samples, although the specific methodologies and handling procedures were not described, limiting interpretability (39). In case of BALf collection, the volume of sterile saline used can substantially affect measured TSLP concentrations. When not corrected for dilution, results of TSLP measurements may not accurately reflect local inflammatory activity, and do not allow for direct comparison across studies. Among the studies included in our systematic review, none reported correction for protein concentration or other normalization approaches in BALf. Bronchial biopsy also presents limitations, as it reflects TSLP expression only at the site of tissue sampling and may not accurately represent the overall inflammatory status of the lung.

Several studies have reported a correlation between higher TSLP concentrations in various biological specimens and airflow obstruction measured by spirometry. The strength of this association varied across studies, with stronger correlations observed in smaller cohorts and weaker in larger populations, potentially reflecting differences in sample size (Table 3).

The study by Ying et al. (36) reported a strong correlation between TSLP expression in bronchial biopsy and FEV1; however, due to a small study population (*n* = 13) and TSLP measured only in epithelium and submucosa, the findings may not be generalizable.

In our study, meta-analysis demonstrated high heterogeneity, with age identified as a potential factor that might contribute to high heterogeneity, based on subgroup analysis. Similarly, in the meta-analysis of 14 studies assessing TSLP concentration in the blood of patients with atopic dermatitis, the heterogeneity was also high (*I*^2^ = 97.46%) (65). Meta-regression analysis showed that mean age and proportion of males among atopic dermatitis patients had a significant impact on heterogeneity, while sample size, year of publication, and disease activity had no influence. Despite this, sources of high heterogeneity remained difficult to explain.

Methodological inconsistencies, combined with diverse patient characteristics, contribute to the variability in TSLP levels across studies, making direct comparisons challenging and leading to limitations of the conclusions.

### 4.6 Implications for clinical practice and policy

Increased concentration and expression of TSLP in blood and airway specimens in asthmatic patients compared with healthy controls highlight its potential utility as a biomarker for asthma management. Additionally, several studies demonstrate a negative correlation of TSLP levels with lung function parameters, suggesting TSLP as a potential indicator of airway inflammation. However, the collection of airway specimens, such as bronchial biopsies or bronchoalveolar lavage fluid (BALf), is not routinely performed during asthma management.

Considering current biological treatment options, the question is raised whether baseline TSLP levels are predictive of the response to anti-TSLP monoclonal antibodies. A *post hoc* analysis of the PATHWAY randomized clinical trial (RCT) showed that tezepelumab is effective in reducing exacerbation rates regardless of baseline serum TSLP concentration in patients with severe, uncontrolled asthma (54).

### 4.7 Future research directions

The integration of TSLP in clinical practice would need standardized protocols for TSLP measurement, including standardized sensitivity and specificity threshold values of detection assays. Normal values for serum TSLP concentration levels ought to be defined in a healthy population.

Currently available ELISA kits measure total TSLP expression, not distinguishing between long and short isoforms of TSLP. Only one included study in the systematic review assessed expression of sfTSLP and lfTSLP using RT-PCR; however, it examines the association of TSLP expression with TSLP SNPs and impact on asthma prevalence (47). We found no study analyzing the relation of expression of lfTSLP with airway inflammation and clinical correlates in asthma. Considering potential distinct roles of lfTSLP and sfTSLP in airway inflammation, future studies should analyze TSLP isoforms separately or analyze only the long form of TSLP.

Moreover, TSLP can be detected with greater accuracy and precision using novel diagnostic tools, e.g., electrochemiluminescence (66) or Meso Scale Discovery (MSD). MSD provides an additional signal enhancement and sensitivity and has a broader dynamic range compared with other conventional electrochemiluminescence assays.

## 5 Conclusions

Our meta-analysis suggests that blood TSLP concentration is higher in asthmatics compared with healthy controls, but the small number of studies and their high heterogeneity reduce the reliability of this finding. The results of TSLP expression in airway specimens were diverse. Standardization and validation of TSLP assays are essential before considering their clinical implementation.

While TSLP has been extensively studied in asthma, unresolved research gaps and substantial variability across studies limit the strength of current conclusions. Further studies are needed to better characterize the relationship between serum TSLP and local airway TSLP expression.

## Data Availability

The original contributions presented in the study are included in the article/Supplementary Material; further inquiries can be directed to the corresponding authors.
