# Supplementary material for: Thymic stromal lymphopoietin expression in different biological specimens in asthma: a systematic review and meta-analysis
Source: Front Allergy. 2025 Nov 20;6:1703989. doi: 10.3389/falgy.2025.1703989 (PMC12675202; doi:10.3389/falgy.2025.1703989)
Supplement: Supplementary file 1 [file Table1.docx]

**Supplementary Materials**

**1. Systematic search Strategy**

A search was performed on four databases including:

**1) PubMed -** (Thymic Stromal Lymphopoietin OR TSLP) AND (asthma or asthmatic)

**2) Embase** - ('Thymic Stromal Lymphopoietin' OR 'tslp') AND ('asthma' OR 'asthmatic') AND [embase/lim NOT ([embase/lim AND [medline/lim)

**3) Cochrane** - (Thymic Stromal Lymphopoietin OR TSLP) AND (asthma OR asthmatic) in All Text

**4) Web of science** - ALL=((Thymic Stromal Lymphopoietin OR TSLP) AND (asthma*))

First search was performed on 22 April 2024, the up-date search was performed 6 months after, on 22 October 2024.

**2. Specification of Enzyme-linked immunosorbent assays (ELISA) kits used in included studies**

|  | **Study** **ID** | **Analysed specimen** | **Assay** **type** | **Manufacturer** | **Sensitivity** | **Minimal detection level** | **Detection range** |
| --- | --- | --- | --- | --- | --- | --- | --- |
| 1. | Ying et al. 2008 (1) | BALf | ELISA | Novartis | 1.0 pg/ml | - | - |
| 2. | Versuluis et al. 2008 (2) | IS | ELISA | R&D Systems | - | - | - |
| 3. | Semlali et al. 2010 (3) | bronchial biopsy | ELISA | R&D Systems | - | 3.46 pg/mL | - |
| 4. | Nguyen et al. 2010 (4) | BALf | ELISA | eBioscience | - | - | - |
| 5. | Kaur et al. 2012 (5) | IS | ELISA | R&D Systems | - | - | - |
| 6. | Koussih et al. 2012 (6) | serum | ELISA | R&D Systems, Minneapolis, MN | 7 .8 pg/ml | - | - |
| 7. | Manthei et al. 2014 (7) | nasal lavage fluid | ELISA | ND | - | 10.5 pg/mL | - |
| 8. | Chauhan et al. 2015 (8) | plasma | ELISA | RayBiotech, Inc., Norcross, GA | - | - | - |
| 9. | Han et al. 2016 (9) | blood | ELISA | R&D Systems | - | - | - |
| 10. | Lai et al. 2016 (10) | serum | ELISA | Uscn Life Science Inc. Wuhan | - | 5.7 pg/ml, | - |
| 11. | Berraïes et al. 2016 (11) | IS, serum | ELISA | R&D, Minneapolis, MN | - | 32 pg/ml | - |
| 12. | Glück et al. 2017 (12) | EBC, serum | ELISA | R&D Systems, USA | 9.87 pg/ml | - | - |
| 13. | Górska et al. 2016 (13) | IS, EBC, serum | ELISA | R&D Systems, Minneapolis, Cat. No DTSLP0 | 9.87 pg/ml | - | 31.3-2000 pg/ml |
|  |  |  |  | EIAab WUHAN, CHINA, Cat. No E1320h | 5.6 pg/ml | - | 31.2-2000 pg/ml |
| 14. | Lin et al. 2016 (14) | serum | ELISA | R&D Systems, Minneapolis, MN | - | - | - |
| 15. | Chai et al. 2017 (15) | serum | ELISA | ND | - | - | - |
| 16. | Wang et al. 2018 (16) | plasma | ELISA | CUSABIO, Wuhan, China | - | - | - |
| 17. | Li et al. 2018 (17) | BALf | ELISA | Novartis | - | 1 pg/ml | - |
| 18. | Liu et al. 2018 (18) | BALf | ELISA | eBioscience, Inc. | - | 15.6 pg/ml | - |
| 19. | Kalinauskaite-Zukauske et al. 2019 (19) | serum | ELISA | R&D Systems, USA | - | 7.8 pg/ml | - |
| 20. | Ma et al. 2019 (20) | serum | ELISA | Abcam Co., Cambridge, UK | - | - | - |
| 21. | Majewski et al. 2019 (21) | EBC | ELISA | R&D Systems, USA | - | - | - |
| 22. | Nejman-Gryz et al. 2020 (22) | IS, serum | ELISA | Invitrogen, Carlsbad, CA, USA | 3 pg/ml | - | 3.3-800 pg/ml |
| 23. | Kozlova et al. 2020 (23) | serum | ELISA | R&D Systems, USA | - | - | - |
| 24. | Zhang et al. 2021 (24) | BALf | ELISA | R&D Systems | - | - | - |
| 25. | Aluraiki et al. 2022 (25) | plasma | ELISA | R&D Systems, Cat. no. DTSLP0 | - | - | - |
| 26. | Basu et al. 2022 (26) | serum | MSD | - | - | - | - |
| 27. | Murrison et al. 2022 (27) | plasma | ELISA | Thermo Fisher Scientific, Cat. no. #88-7497 | - | 8 pg/ml | - |
| 28. | Türk et al. 2022 (28) | serum | ELISA | Boster Biological Technology, Pleasanton CA, USA | - | - | - |
| 29. | Vrsalović et al. 2022 (29) | serum | ELISA | Abcam, ab155444 | - | - | - |
| 30. | Andreasson et al. 2023 (30) | serum | MSD | Cat. no. #K151D3S | - | - | 9.1-70,000 fg/mL |
| 31. | Ibrahim et al. 2023 (31) | plasma | MSD | Meso Scale Diagnostics, Rockville, Massachusetts, USA | - | - | 0.13-100 pg/ml |
| 32. | Doulatpanah et al. 2023 (32) | nasal fluid, serum | MSD | Luminex®, LXSAHM, RnDSystems, MN, USA | - | - | - |

**Supplementary Table 1. Specification of Enzyme-linked immunosorbent assays (ELISA) kits used in included studies**. MSD - MesoScale Discovery electrochemiluminescent multiplex immunoassay,

**3. Sources of funding and conflict of interest in included studies**

|  | **Study** **ID** | **Conflict of interest** | **Source of funding** |
| --- | --- | --- | --- |
| 1. | Ying et al. 2005 (33) | No conflict of interest | Partially supported by the Central Research Fund of University of London, U.K., Asthma U.K., and the Department of Asthma, Allergy and Respiratory Science, Guy’s, King’s and St. Thomas’ School of Medicine, London, U.K. D.R. is supported by a Wellcome Trust Research Leave Award for Clinical Academics |
| 2. | Ying et al. 2008 (1) | No conflict of interest | Supported in part by the Central Research Fund of the University of London, Asthma U.K., and Department of Asthma, Allergy and Respiratory Science, King’s College London. The authors acknowledge financial support from the Department of Health via the National Institute for Health Research (NIHR) comprehensive Biomedical Research Centre award to Guy’s & St Thomas’ NHS Foundation Trust in partnership with King’s College London. |
| 3. | Versluis et al. 2008 (2) | ND | GlaxoSmithKline, Graduate School for Drug Exploration (GUIDE), Stichting Astma Bestrijding |
| 4. | Semlali et al. 2010 (3) | No conflict of interest | Supported by the Canadian Institutes of Health Research (CIHR). Dr Chakir is the recipient of Chercheur-Boursier Awards from the Fonds de la Recherche en Sante ́ du Quebec; Dr Semlali is the recipient of a GSK/CIHR fellowship |
| 5. | Nguyen et al. 2010 (4) | No conflict of interest | Grants from the Mary Hewitt Loveless Foundation, the Parker B. Francis Foundation, and the American Academy of Allergy, Asthma, and Immunology. |
| 6. | Shikotra et al. 2011 (34) | D. F. Choy, A. R. Abbas, C. D. Austin, J. Jackman, L. C. Wu, and J. R. Arron are employees of Genentech, Inc. L. G. Heaney has received travel and accommodation support to attend meetings from AstraZeneca, Chiesi, Novartis, GlaxoSmithKline, and Teva UK; has received research support from GlaxoSmithKline, Genentech, Inc, MedImmune, and Novartis UK; and has served on advisory boards for or received speakers’ honoraria from GlaxoSmithKline, Merck Sharpe & Dohme, Nycomed, Novartis, and AstraZeneca. P. Bradding has received research support from Genentech, Inc. | The Institute for Lung Health, Department of Infection, Immunity and Inflammation, University of Leicester, Leicester, United Kingdom, and the Centre for Infection and Immunity, Health Sciences Building, Queens University Belfast, were supported by grants from Genentech, Inc, South San Francisco, Calif. Research at the Institute for Lung Health, Department of Infection, Immunity and Inflammation, University of Leicester, Leicester, United Kingdom, was conducted in laboratories partially funded by ERDF no. 05567. |
| 7. | Kaur et al. 2012 (5) | Dr Brightling has received consultancy fees and research funding from AstraZeneca, MedImmune LLC, GlaxoSmithKline, Chiesi Ltd, Hoffmann-La Roche Inc, and Novartis AG. | Supported by GlaxoSmithKline. Dr Brightling was supported by a Wellcome Senior Clinical Fellowship [082265 |
| 8. | Koussih et al. 2012 (6) | No conflict of interest | Grants from the Canadian Institutes of Health Research (MOP: 53104) to ASG and AB. ASG is supported by a Manitoba Research Chair from the Manitoba Health Research Council |
| 9. | Wu et al. 2012 (35) | No conflict of interest | ND |
| 10. | Wu et al. 2013 (36) | No conflict of interest | Supported by National Nature Science Foundation of China (NSFC) (NSFC for LD, Grant No: 81270072), Natural Science Funding committee of Shandong province (SDNSF) (Grant No: ZR2011HM020) and Longhua Medical Project (RC) |
| 11. | Cheng et al. 2014 (37) | ND | Supported by National Natural Science Foundation of China (grants 81170022 and 30700244), Scientific Research Foundation for the Returned Overseas Chinese Scholars of State Education Ministry grant (2008890), National Key Technology R&D Program of the 12th Five-year Development Plan (2012BAI05B01), the UCSF Sandler Asthma Basic Research Center, and the National Institutes of Health (grants AI1077439 and HL099101). |
| 12. | Manthei et al. 2014 (7) | SKM, JEG, NNJ, and LCD are PI or co-PI on NIH grants. SKM is a consultant for TEVA; JEG is a consultant for GlaxoSmithKline, Biota, Centocor, Boehringer Ingelheim, MedImmune, Theraclone, Merck and Gilead; NNJ is a consultant for Boston Scientific and Genentech; LCD is a consultant for Novartis | Supported by the Clinical and Translational Science Award (CTSA) program, through the NIH National Center for Advancing Translational Sciences (NCATS), grant UL1TR000427, as well as National Institutes of Health Grants R01 HL080412, R01 HL115118, P01 HL088594, U19 AI070503, K23 HL081492, and T32 GM008692. |
| 13. | Bleck et al. 2015 (38) | 3 authors declare conflict of interest (M. Liu received research support from the Centers for Disease Control and Prevention;  D. B. Tse received royalties from UC Berkeley;  J. Reibman received research support from the National Institutes of Health and has received funds from Novartis, Astra-Zeneca, and Med- Immune.) | National Institutes of Health (NIH)/National Institute of Environmental Health Sciences (NIEHS) grant 5R01ES010187 (to J.R.), CTSI grant NIH/NCRR 1UL1RR029893-0109, the Stony Wold-Herbert Fund, NIH/NIEHS grant 5T32ES007267, 1R01 HL095764-01 (to G.G.), the Colton Family Foundation, Ginsberg/Kanengiser, and the Arthur Goldfarb Foundations. |
| 14. | Chauhan et al. 2015 (8) | No conflict of interest | ND |
| 15. | Han et al. 2016 (9) | No conflict of interest | ND |
| 16. | Lai et al. 2016 (10) | No conflict of interest | Grant of the Medical Scientific Research Project of Guangdong Province, China (No. A2012430) |
| 17. | Berraïes et al. 2016 (11) | ND | Supported by Ministry of Higher Education and Scientific Research of Tunisia |
| 18. | Glück et al. 2016 (12) | ND | This study was supported by grant KNW-135-P/2/0 from the Medical University of Silesia. |
| 19. | Górska et al. 2016 (13) | No conflict of interest | ND |
| 20. | Lin et al. 2016 (14) | No conflict of interest | Supported by the Ministry of Science and Technology of Taiwan (MOST103-2314-B-038-018) |
| 21. | Chai et al. 2017 (15) | No conflict of interest | No funding |
| 22. | Wang et al. 2018 (16) | No conflict of interest | Supported by the National Natural Science Foundation of China (grant number 81650003) and the Health and Family Planning Commission of Chongqing City (grant numbers 2016HBRC004), |
| 23. | Li et al. 2018 (17) | No conflict of interest | No source of funding reported; We thank Novartis Institute of Biomedical Research, Horsham, U.K.) for technical support |
| 24. | Liu et al. 2018 (18) | M. M. Gorska receives grant support from the National Institutes of Health; R. J. Martin serves as a consultant for AstraZeneca, Teva Pharmaceuticals, PMD Healthcare, MedImmune, and Genentech; grant support from MedImmune, Chiesi Famaceutci SpA, and the National Heart, Lung, and Blood Institute; royalties from UpToDate; and travel support from the Respiratory Effectiveness Group | The work was supported by NIH grants RO1 AI091614, HL126895, AI102943 and HL126895. |
| 25. | Kalinauskaite‐Zukauske et al. 2019 (19) | No conflict of interest | ND |
| 26. | Ma et al. 2019 (20) | No conflict of interest | No funding |
| 27. | Majewski et al. 2019 (21) | No conflict of interest | Funded by the Medical University of Lodz and regular finances of the Department of Pneumology and Allergy 503/1‐151‐03/503‐11‐002‐18 |
| 28. | Nejman-Gryz et al. 2020 (22) | No conflict of interest | No funding |
| 29. | Kozlova et al. 2020 (23) | No conflict of interest | ND |
| 30. | Kim et al. 2020 (39) | No conflict of interest | Supported by a grant from the Basic Science Research Program through the National Research Foundation of Korea funded by the Ministry of Education (NFR 2017R1C1B5076565) and a grant from the Hallym University Medical Center Research Fund (01-2012-12) |
| 31. | Zhang et al. 2021 (24) | No conflict of interest | Supported by National Natural Science Foundation of China (grant nos. 91742108, 81670019, 81800026, 81600023), National Key Research and Development Program of China (grant no. 2016YFC1304400), and Hubei Province Natural Science Foun- dation (grant no. 2017CFA016) |
| 32. | Alturaiki et al. 2022 (25) | No conflict of interest | Deanship of Scientific Research at Maj- maah University for funding the study under project number [R- 2022-211 |
| 33. | Basu et al. 2022 (26) | No conflict of interest | Ulla Damgaard Munk is acknowledged for skilled technical assistance and Thermo Fisher Scientific for their generous contribution with the IgE analysis |
| 34. | Murrison et al. 2022 (27) | No conflict of interest | Supported by National Institute of Allergy and Infectious Diseases grant R01AI127392 (to G.K.K.H., L.B.M., J.M.B., L.J.M., R.K.) and U19AI1070235 (to G.K.K.H., J.M.B., L.J.M.) |
| 35. | Türk et al. 2022 (28) | ND | ND |
| 36. | Vrsalović et al. 2022 (29) | No conflict of interest | No funding |
| 37. | Andreasson et al. 2023 (30) | M. Hvidtfeldt has received consulting fees from Novartis and speaker fees from AstraZeneca, GlaxoSmithKline, and BIRK; A. von Bülow has received consulting fees from Novartis; speaker fees from Novartis, GlaxoSmithKline, and AstraZeneca; and travel grants from AstraZeneca; and has served on advisory boards for AstraZeneca and Novartis. L. Uller has received consulting fees/honoraria from AstraZeneca; C. Porsbjerg has attended advisory boards for AstraZeneca, Novartis, TEVA, and Sanofi-Genzyme; has given lectures at meetings supported by AstraZeneca, Novartis, TEVA, Sanofi-Genzyme, Chiesi, and GlaxoSmithKline; has taken part in clinical trials sponsored by AstraZeneca, Novartis, MSD, Sanofi-Genzyme, GlaxoSmithKline, and Novartis; and has received educational and research grants from AstraZeneca, Novartis, TEVA, GlaxoSmithKline, ALK, and Sanofi-Genzyme; A. Sverrild has attended advisory boards for AstraZeneca, GlaxoSmithKline, and Sanofi-Regeneron; has given lectures at meetings supported by AstraZeneca and Chiesi; and has received educational and research grants from AstraZeneca. | Supported by internal funds from Bispebjerg Hospital, Copenhagen, Denmark |
| 38. | Ibrahim et al. 2023 (31) | No conflict of interest | Funded by the French National Research Program for Environmental and Occupational Health of Anses (EST/2017/1/158) and the region Hauts-de-France |
| 39. | Doulatpanah et al. 2023 (32) | No conflict of interest | Office of Scientific Research Projects of Ege University with the project ID 18-TIP-022 |
| 40. | Połomska et al. 2024 (40) | No conflict of interest | Funded by Wroclaw Medical University, Grant number SUBZ.A220.22.072. |

**Supplementary Table 2. Potential conflicts of interests and sources of funding of included studies.**

ND - no data,

**4. PRISMA 2020 checklist (41)**

|  |  | Reporting Item | Page Number |
| --- | --- | --- | --- |
| **Title** |  |  |  |
| Title | [#1](https://www.goodreports.org/reporting-checklists/prisma/info/#1) | Identify the report as a systematic review | 1 |
| **Abstract** |  |  |  |
| Abstract | [#2](https://www.goodreports.org/reporting-checklists/prisma/info/#2) | Report an abstract addressing each item in the PRISMA 2020 for Abstracts checklist | 1,2 |
| **Introduction** |  |  |  |
| Background/rationale | [#3](https://www.goodreports.org/reporting-checklists/prisma/info/#3) | Describe the rationale for the review in the context of existing knowledge | 3 |
| Objectives | [#4](https://www.goodreports.org/reporting-checklists/prisma/info/#4) | Provide an explicit statement of the objective(s) or question(s) the review addresses | 4 |
| **Methods** |  |  |  |
| Eligibility criteria | [#5](https://www.goodreports.org/reporting-checklists/prisma/info/#5) | Specify the inclusion and exclusion criteria for the review and how studies were grouped for the syntheses | 4,5 |
| Information sources | [#6](https://www.goodreports.org/reporting-checklists/prisma/info/#6) | Specify all databases, registers, websites, organisations, reference lists, and other sources searched or consulted to identify studies. Specify the date when each source was last searched or consulted | 4 |
| Search strategy | [#7](https://www.goodreports.org/reporting-checklists/prisma/info/#7) | Present the full search strategies for all databases, registers, and websites, including any filters and limits used | Supplementary materials, page 1 |
| Selection process | [#8](https://www.goodreports.org/reporting-checklists/prisma/info/#8) | Specify the methods used to decide whether a study met the inclusion criteria of the review, including how many reviewers screened each record and each report retrieved, whether they worked independently, and, if applicable, details of automation tools used in the process | 5 |
| Data collection process | [#9](https://www.goodreports.org/reporting-checklists/prisma/info/#9) | Specify the methods used to collect data from reports, including how many reviewers collected data from each report, whether they worked independently, any processes for obtaining or confirming data from study investigators, and, if applicable, details of automation tools used in the process | 5,6 |
| Data items | [#10a](https://www.goodreports.org/reporting-checklists/prisma/info/#10a) | List and define all outcomes for which data were sought. Specify whether all results that were compatible with each outcome domain in each study were sought (for example, for all measures, time points, analyses), and, if not, the methods used to decide which results to collect | 6 |
| Study risk of bias assessment | [#11](https://www.goodreports.org/reporting-checklists/prisma/info/#11) | Specify the methods used to assess risk of bias in the included studies, including details of the tool(s) used, how many reviewers assessed each study and whether they worked independently, and, if applicable, details of automation tools used in the process | 6 |
| Effect measures | [#12](https://www.goodreports.org/reporting-checklists/prisma/info/#12) | Specify for each outcome the effect measure(s) (such as risk ratio, mean difference) used in the synthesis or presentation of results | 6 |
| Synthesis methods | [#13a](https://www.goodreports.org/reporting-checklists/prisma/info/#13a) | Describe the processes used to decide which studies were eligible for each synthesis (such as tabulating the study intervention characteristics and comparing against the planned groups for each synthesis (item #5)) | 6 |
| Synthesis methods | [#13b](https://www.goodreports.org/reporting-checklists/prisma/info/#13b) | Describe any methods required to prepare the data for presentation or synthesis, such as handling of missing summary statistics or data conversions | 6 |
| Synthesis methods | [#13c](https://www.goodreports.org/reporting-checklists/prisma/info/#13c) | Describe any methods used to tabulate or visually display results of individual studies and syntheses | 6 |
| Synthesis methods | [#13d](https://www.goodreports.org/reporting-checklists/prisma/info/#13d) | Describe any methods used to synthesise results and provide a rationale for the choice(s). If meta-analysis was performed, describe the model(s), method(s) to identify the presence and extent of statistical heterogeneity, and software package(s) used | 6 |
| Synthesis methods | [#13e](https://www.goodreports.org/reporting-checklists/prisma/info/#13e) | Describe any methods used to explore possible causes of heterogeneity among study results (such as subgroup analysis, meta-regression) | 6 |
| Synthesis methods | [#13f](https://www.goodreports.org/reporting-checklists/prisma/info/#13f) | Describe any sensitivity analyses conducted to assess robustness of the synthesised results | 6 |
| Reporting bias assessment | [#14](https://www.goodreports.org/reporting-checklists/prisma/info/#14) | Describe any methods used to assess risk of bias due to missing results in a synthesis (arising from reporting biases) | 6 |
| Certainty assessment | [#15](https://www.goodreports.org/reporting-checklists/prisma/info/#15) | Describe any methods used to assess certainty (or confidence) in the body of evidence for an outcome | 6 |
| Data items | [#10b](https://www.goodreports.org/reporting-checklists/prisma/info/#10b) | List and define all other variables for which data were sought (such as participant and intervention characteristics, funding sources). Describe any assumptions made about any missing or unclear information | 6 |
| **Results** |  |  |  |
| Study selection | [#16a](https://www.goodreports.org/reporting-checklists/prisma/info/#16a) | Describe the results of the search and selection process, from the number of records identified in the search to the number of studies included in the review, ideally using a flow diagram (http://www.prisma-statement.org/PRISMAStatement/FlowDiagram) | 7, 19 |
| Study selection | [#16b](https://www.goodreports.org/reporting-checklists/prisma/info/#16b) | Cite studies that might appear to meet the inclusion criteria, but which were excluded, and explain why they were excluded | 19 |
| Study characteristics | [#17](https://www.goodreports.org/reporting-checklists/prisma/info/#17) | Cite each included study and present its characteristics | 8, 23-29 |
| Risk of bias in studies | [#18](https://www.goodreports.org/reporting-checklists/prisma/info/#18) | Present assessments of risk of bias for each included study | 9, 30-31 |
| Results of individual studies | [#19](https://www.goodreports.org/reporting-checklists/prisma/info/#19) | For all outcomes, present for each study (a) summary statistics for each group (where appropriate) and (b) an effect estimate and its precision (such as confidence/credible interval), ideally using structured tables or plots | 23-29 |
| Results of syntheses | [#20a](https://www.goodreports.org/reporting-checklists/prisma/info/#20a) | For each synthesis, briefly summarise the characteristics and risk of bias among contributing studies | 9,30-31 |
| Results of syntheses | [#20b](https://www.goodreports.org/reporting-checklists/prisma/info/#20b) | Present results of all statistical syntheses conducted. If meta-analysis was done, present for each the summary estimate and its precision (such as confidence/credible interval) and measures of statistical heterogeneity. If comparing groups, describe the direction of the effect | 20-21 |
| Results of syntheses | [#20c](https://www.goodreports.org/reporting-checklists/prisma/info/#20c) | Present results of all investigations of possible causes of heterogeneity among study results | 20, 21 |
| Results of syntheses | [#20d](https://www.goodreports.org/reporting-checklists/prisma/info/#20d) | Present results of all sensitivity analyses conducted to assess the robustness of the synthesised results | 20 |
| Risk of reporting biases in syntheses | [#21](https://www.goodreports.org/reporting-checklists/prisma/info/#21) | Present assessments of risk of bias due to missing results (arising from reporting biases) for each synthesis assessed | 8 |
| Certainty of evidence | [#22](https://www.goodreports.org/reporting-checklists/prisma/info/#22) | Present assessments of certainty (or confidence) in the body of evidence for each outcome assessed | 8 |
| **Discussion** |  |  |  |
| Results in context | [#23a](https://www.goodreports.org/reporting-checklists/prisma/info/#23a) | Provide a general interpretation of the results in the context of other evidence | 13,14 |
| Limitations of included studies | [#23b](https://www.goodreports.org/reporting-checklists/prisma/info/#23b) | Discuss any limitations of the evidence included in the review | 15 |
| Limitations of the review methods | [#23c](https://www.goodreports.org/reporting-checklists/prisma/info/#23c) | Discuss any limitations of the review processes used | 15 |
| Implications | [#23d](https://www.goodreports.org/reporting-checklists/prisma/info/#23d) | Discuss implications of the results for practice, policy, and future research | 16 |
| **Other information** |  |  |  |
| Registration and protocol | [#24a](https://www.goodreports.org/reporting-checklists/prisma/info/#24a) | Provide registration information for the review, including register name and registration number, or state that the review was not registered | 3 |
| Registration and protocol | [#24b](https://www.goodreports.org/reporting-checklists/prisma/info/#24b) | Indicate where the review protocol can be accessed, or state that a protocol was not prepared | 4 |
| Registration and protocol | [#24c](https://www.goodreports.org/reporting-checklists/prisma/info/#24c) | Describe and explain any amendments to information provided at registration or in the protocol | 4 |
| Support | [#25](https://www.goodreports.org/reporting-checklists/prisma/info/#25) | Describe sources of financial or non-financial support for the review, and the role of the funders or sponsors in the review | 18 |
| Competing interests | [#26](https://www.goodreports.org/reporting-checklists/prisma/info/#26) | Declare any competing interests of review authors | 18 |
| Availability of data, code, and other materials | [#27](https://www.goodreports.org/reporting-checklists/prisma/info/#27) | Report which of the following are publicly available and where they can be found: template data collection forms; data extracted from included studies; data used for all analyses; analytic code; any other materials used in the review | 18 |

**References**

1. Ying S, O'Connor B, Ratoff J, Meng Q, Fang C, Cousins D, et al. Expression and cellular provenance of thymic stromal lymphopoietin and chemokines in patients with severe asthma and chronic obstructive pulmonary disease. J Immunol. 2008;181(4):2790-8.

2. Versluis M, van den Berge M, Timens W, Luijk B, Rutgers B, Lammers JWJ, et al. Allergen inhalation decreases adenosine receptor expression in sputum and blood of asthma patients. Allergy. 2008;63(9):1186-94.

3. Semlali A, Jacques E, Koussih L, Gounni AS, Chakir J. Thymic stromal lymphopoietin-induced human asthmatic airway epithelial cell proliferation through an IL-13-dependent pathway. J Allergy Clin Immunol. 2010;125(4):844-50.

4. Nguyen KD, Vanichsarn C, Nadeau KC. TSLP directly impairs pulmonary Treg function: association with aberrant tolerogenic immunity in asthmatic airway. Allergy Asthma Clin Immunol. 2010;6(1):4.

5. Kaur D, Doe C, Woodman L, Heidi Wan WY, Sutcliffe A, Hollins F, et al. Mast cell-airway smooth muscle crosstalk: the role of thymic stromal lymphopoietin. Chest. 2012;142(1):76-85.

6. Koussih L, Ali A, Shan L, Becker A, Gounni AS. Serum level of thymic stromal lymphopoietin in allergic asthmatic children. Clin Immunol. 2012;145(2):92-3.

7. Manthei DM, Schwantes EA, Mathur SK, Guadarrama AG, Kelly EA, Gern JE, et al. Nasal lavage VEGF and TNF-α levels during a natural cold predict asthma exacerbations. Clin Exp Allergy. 2014;44(12):1484-93.

8. Chauhan A, Singh M, Agarwal A, Paul N. Correlation of TSLP, IL-33, and CD4 + CD25 + FOXP3 + T regulatory (Treg) in pediatric asthma. J Asthma. 2015;52(9):868-72.

9. Han XM, Cheng YY, Gong YF, Jiang MM. The correlation between children's status asthmatics and interstitial lung disease. Eur Rev Med Pharmacol Sci. 2016;20(22):4761-5.

10. Lai T, Wu D, Li W, Chen M, Yi Z, Huang D, et al. Interleukin-31 expression and relation to disease severity in human asthma. Sci Rep. 2016;6:22835.

11. Berraïes A, Hamdi B, Ammar J, Hamzaoui K, Hamzaoui A. Increased expression of thymic stromal lymphopoietin in induced sputum from asthmatic children. Immunol Lett. 2016;178:85-91.

12. Glück J, Rymarczyk B, Kasprzak M, Rogala B. Increased Levels of Interleukin-33 and Thymic Stromal Lymphopoietin in Exhaled Breath Condensate in Chronic Bronchial Asthma. Int Arch Allergy Immunol. 2016;169(1):51-6.

13. Górska K, Nejman-Gryz P, Paplińska-Goryca M, Proboszcz M, Krenke R. Comparison of Thymic Stromal Lymphopoietin Concentration in Various Human Biospecimens from Asthma and COPD Patients Measured with Two Different ELISA Kits. Adv Exp Med Biol. 2017;955:19-27.

14. Lin SC, Huang JJ, Wang JY, Chuang HC, Chiang BL, Ye YL. Upregulated thymic stromal lymphopoietin receptor expression in children with asthma. Eur J Clin Invest. 2016;46(6):511-9.

15. Chai R, Liu B, Qi F. The significance of the levels of IL-4, IL-31 and TLSP in patients with asthma and/or rhinitis. Immunotherapy. 2017;9(4):331-7.

16. Wang J, Lv H, Luo Z, Mou S, Liu J, Liu C, et al. Plasma YKL-40 and NGAL are useful in distinguishing ACO from asthma and COPD. Respir Res. 2018;19(1):47.

17. Li Y, Wang W, Lv Z, Li Y, Chen Y, Huang K, et al. Elevated Expression of IL-33 and TSLP in the Airways of Human Asthmatics In Vivo: A Potential Biomarker of Severe Refractory Disease. J Immunol. 2018;200(7):2253-62.

18. Liu S, Verma M, Michalec L, Liu W, Sripada A, Rollins D, et al. Steroid resistance of airway type 2 innate lymphoid cells from patients with severe asthma: The role of thymic stromal lymphopoietin. J Allergy Clin Immunol. 2018;141(1):257-68.e6.

19. Kalinauskaite-Zukauske V, Janulaityte I, Januskevicius A, Malakauskas K. Serum levels of epithelial-derived mediators and interleukin-4/interleukin-13 signaling after bronchial challenge with Dermatophagoides pteronyssinus in patients with allergic asthma. Scand J Immunol. 2019;90(5):e12820.

20. Ma SL, Zhang L. Elevated serum OX40L is a biomarker for identifying corticosteroid resistance in pediatric asthmatic patients. BMC Pulm Med. 2019;19(1):66.

21. Majewski S, Tworek D, Szewczyk K, Kurmanowska Z, Antczak A, Górski P, et al. Epithelial alarmin levels in exhaled breath condensate in patients with idiopathic pulmonary fibrosis: A pilot study. Clin Respir J. 2019;13(10):652-6.

22. Nejman-Gryz P, Górska K, Paplińska-Goryca M, Proboszcz M, Krenke R. Periostin and Thymic Stromal Lymphopoietin-Potential Crosstalk in Obstructive Airway Diseases. J Clin Med. 2020;9(11).

23. Kozlova Y, Frolova E, Uchevatkina A, Filippova L, Aak O, Burygina E, et al. Diagnostic markers of allergic bronchopulmonary aspergillosis in patients with severe asthma. Mycoses. 2020;63(6):596-603.

24. Zhang K, Feng Y, Liang Y, Wu W, Chang C, Chen D, et al. Epithelial miR-206 targets CD39/extracellular ATP to upregulate airway IL-25 and TSLP in type 2-high asthma. JCI Insight. 2021;6(11).

25. Alturaiki W. High plasma levels of the TSLP cytokine in Saudi patients with chronic stable asthma. Journal of King Saud University Science. 2022;34(7).

26. Basu MN, Mortz CG, Jensen TK, Barington T, Lambertsen KL, Halken S. Biomarkers in asthma in the context of atopic dermatitis in young children. Pediatr Allergy Immunol. 2022;33(7):e13823.

27. Murrison LB, Ren X, Preusse K, He H, Kroner J, Chen X, et al. TSLP disease-associated genetic variants combined with airway TSLP expression influence asthma risk. J Allergy Clin Immunol. 2022;149(1):79-88.

28. Türk M, Yılmaz İ, Gökahmetoğlu S, Koç AN. Stable and exacerbation period serum cytokine and periostin levels of the five distinct phenotypes of severe asthma. Turk J Med Sci. 2022;52(4):1148-59.

29. Vrsalović R, Korošec P, Štefanović IM, Bidovec-Stojkovič U, Čičak B, Harjaček M, et al. Value of thymic stromal lymphopoietin as a biomarker in children with asthma. Respir Med. 2022;193:106757.

30. Andreasson LM, Dyhre-Petersen N, Hvidtfeldt M, Jørgensen G, Von Bülow A, Klein DK, et al. Airway hyperresponsiveness correlates with airway TSLP in asthma independent of eosinophilic inflammation. J Allergy Clin Immunol. 2023.

31. Ibrahim B, Achour D, Zerimech F, de Nadai P, Siroux V, Tsicopoulos A, et al. Plasma thymic stromal lymphopoietin (TSLP) in adults with non-severe asthma: the EGEA study. Thorax. 2023;78(2):207-10.

32. Doulatpanah M, Kocamanoğlu M, Sözmen EY, Öztürk GK, Demir E, Gülen F, et al. Nasal fluid sample as a reliable matrix for determination of cytokine levels in childhood asthma. Turkish Journal of Biochemistry. 2023;48(5):507-14.

33. Ying S, O'Connor B, Ratoff J, Meng Q, Mallett K, Cousins D, et al. Thymic stromal lymphopoietin expression is increased in asthmatic airways and correlates with expression of Th2-attracting chemokines and disease severity. J Immunol. 2005;174(12):8183-90.

34. Shikotra A, Choy DF, Ohri CM, Doran E, Butler C, Hargadon B, et al. Increased expression of immunoreactive thymic stromal lymphopoietin in patients with severe asthma. J Allergy Clin Immunol. 2012;129(1):104-11.e1-9.

35. Wu J, Zhao J, Wei Y, Bi W, Dong L, Wang X, et al. Thymic stromal lymphopoietin promotes asthmatic airway remodeling in human lung fibroblast cells through stat3 signaling pathway. European Respiratory Journal. 2012;40.

36. Wu J, Dong F, Wang RA, Wang J, Zhao J, Yang M, et al. Central role of cellular senescence in TSLP-induced airway remodeling in asthma. PLoS One. 2013;8(10):e77795.

37. Cheng D, Xue Z, Yi L, Shi H, Zhang K, Huo X, et al. Epithelial interleukin-25 is a key mediator in Th2-high, corticosteroid-responsive asthma. Am J Respir Crit Care Med. 2014;190(6):639-48.

38. Bleck B, Kazeros A, Bakal K, Garcia-Medina L, Adams A, Liu M, et al. Coexpression of type 2 immune targets in sputum-derived epithelial and dendritic cells from asthmatic subjects. J Allergy Clin Immunol. 2015;136(3):619-27.e5.

39. Kim JH, Jang YS, Kim HI, Park JY, Park SH, Hwang YI, et al. Activation of Transient Receptor Potential Melastatin Family Member 8 (TRPM8) Receptors Induces Proinflammatory Cytokine Expressions in Bronchial Epithelial Cells. Allergy Asthma Immunol Res. 2020;12(4):684-700.

40. Połomska J, Sikorska-Szaflik H, Drabik-Chamerska A, Sozańska B, Dębińska A. Exploring TSLP and IL-33 Serum Levels and Genetic Variants: Unveiling Their Limited Potential as Biomarkers for Mild Asthma in Children. J Clin Med. 2024;13(9).

41. Page MJ, Moher D, Bossuyt PM, Boutron I, Hoffmann TC, Mulrow CD, et al. PRISMA 2020 explanation and elaboration: updated guidance and exemplars for reporting systematic reviews. Bmj. 2021;372:n160.
